# Hypermethylation of *MIR21* in CD4+ T cells from patients with relapsing-remitting multiple sclerosis associates with lower miRNA-21 levels and concomitant up-regulation of its target genes

**DOI:** 10.1177/1352458517721356

**Published:** 2017-08-02

**Authors:** Sabrina Ruhrmann, Ewoud Ewing, Eliane Piket, Lara Kular, Julio Cesar Cetrulo Lorenzi, Sunjay Jude Fernandes, Hiromasa Morikawa, Shahin Aeinehband, Sergi Sayols-Baixeras, Stella Aslibekyan, Devin M Absher, Donna K Arnett, Jesper Tegner, David Gomez-Cabrero, Fredrik Piehl, Maja Jagodic

**Affiliations:** Department of Clinical Neuroscience, Center for Molecular Medicine, Karolinska Institutet, Stockholm, Sweden; Department of Clinical Neuroscience, Center for Molecular Medicine, Karolinska Institutet, Stockholm, Sweden; Department of Clinical Neuroscience, Center for Molecular Medicine, Karolinska Institutet, Stockholm, Sweden; Department of Clinical Neuroscience, Center for Molecular Medicine, Karolinska Institutet, Stockholm, Sweden; Department of Clinical Neuroscience, Center for Molecular Medicine, Karolinska Institutet, Stockholm, Sweden/ Department of Genetics, Medical School of Ribeirão Preto, São Paulo University, Ribeirão Preto, Brazil; Unit of Computational Medicine, Department of Medicine, Solna, Center for Molecular Medicine, Karolinska Institutet, Stockholm, Sweden/ Science for Life Laboratory, Stockholm, Sweden; Unit of Computational Medicine, Department of Medicine, Solna, Center for Molecular Medicine, Karolinska Institutet, Stockholm, Sweden/ Science for Life Laboratory, Stockholm, Sweden; Department of Clinical Neuroscience, Center for Molecular Medicine, Karolinska Institutet, Stockholm, Sweden; Cardiovascular Epidemiology and Genetics Group, Institut Hospital del Mar d’Investigacions Mediques (IMIM), Barcelona, Spain/ Universitat Pompeu Fabra (UPF), Barcelona, Spain; Department of Epidemiology, University of Alabama at Birmingham, Birmingham, AL, USA; HudsonAlpha Institute for Biotechnology, Huntsville, AL, USA; College of Public Health, University of Kentucky, Lexington, KY, USA; Unit of Computational Medicine, Department of Medicine, Solna, Center for Molecular Medicine, Karolinska Institutet, Stockholm, Sweden/ Science for Life Laboratory, Stockholm, Sweden/ Biological and Environmental Sciences and Engineering Division, Computer, Electrical and Mathematical Sciences and Engineering Division, King Abdullah University of Science and Technology (KAUST), Thuwal, Kingdom of Saudi Arabia; Unit of Computational Medicine, Department of Medicine, Solna, Center for Molecular Medicine, Karolinska Institutet, Stockholm, Sweden/ Mucosal & Salivary Biology Division, Dental Institute, King’s College London, London, UK; Department of Clinical Neuroscience, Center for Molecular Medicine, Karolinska Institutet, Stockholm, Sweden; Department of Clinical Neuroscience, Center for Molecular Medicine, Karolinska Institutet, Stockholm, Sweden

**Keywords:** CD4+ T cells, autoimmunity, epigenetics, DNA methylation, microRNAs, multiple sclerosis, relapsing-remitting, miR-21

## Abstract

**Background:**

Multiple sclerosis (MS) is a chronic inflammatory disease of the central nervous system caused by genetic and environmental factors. DNA methylation, an epigenetic mechanism that controls genome activity, may provide a link between genetic and environmental risk factors.

**Objective:**

We sought to identify DNA methylation changes in CD4+ T cells in patients with relapsing-remitting (RR-MS) and secondary-progressive (SP-MS) disease and healthy controls (HC).

**Methods:**

We performed DNA methylation analysis in CD4+ T cells from RR-MS, SP-MS, and HC and associated identified changes with the nearby risk allele, smoking, age, and gene expression.

**Results:**

We observed significant methylation differences in the *VMP1/MIR21* locus, with RR-MS displaying higher methylation compared to SP-MS and HC. *VMP1/MIR21* methylation did not correlate with a known MS risk variant in *VMP1* or smoking but displayed a significant negative correlation with age and the levels of mature miR-21 in CD4+ T cells. Accordingly, RR-MS displayed lower levels of miR-21 compared to SP-MS, which might reflect differences in age between the groups, and healthy individuals and a significant enrichment of up-regulated miR-21 target genes.

**Conclusion:**

Disease-related changes in epigenetic marking of *MIR21* in RR-MS lead to differences in miR-21 expression with a consequence on miR-21 target genes.

## Introduction

Multiple sclerosis (MS) is a chronic inflammatory disease of the central nervous system (CNS) and one of the most common causes of neurological disability in young adults.^1^ The majority of MS patients initially present with the relapsing-remitting disease (RR-MS) characterized by episodes of active disease and periods of clinical inactivity, but most will eventually convert to a secondary-progressive form (SP-MS) characterized by continuous worsening.^1^

MS results from an interplay between genes and environmental factors. The HLA-DRB1*15:01 haplotype has been consistently associated with MS, suggesting critical role of the human leukocyte antigen (HLA) class II molecules in presenting antigens to CD4+ T cells.^2^ The importance of CD4+ T cells has further been supported by their ability to induce MS-like pathology in rodents.^3^ More than 100 non-HLA loci have been associated with MS, jointly pointing to importance of immune-mediated functions such as lymphocyte activation and differentiation.^2,4^ Smoking has also been consistently associated with an increased risk of MS, and carriers of HLA-DRB1*15:01 who are smokers have 14-fold higher risk compared to non-smokers and non-carriers.^5^

Such gene–environment interactions may be mediated through epigenetic mechanisms by which the environment modulates gene expression in a manner that is heritable through cell division. DNA methylation, histone modifications, nuclear complexes, and non-coding RNAs (ncRNAs) comprise main epigenetic mechanisms. DNA methylation, where a methyl group is added to cytosine in the CpG context, is the most studied epigenetic mechanism critical for processes such as genomic imprinting and X chromosome inactivation^6^ and also T-cell activation and differentiation.^7–10^ Two recent studies suggest subtle but numerous DNA methylation changes in CD4+ T cells of RR-MS patients.^11,12^

Besides DNA methylation, gene expression is regulated by ncRNAs like microRNAs (miRNAs) that act post-transcriptionally.^13^ Mature miRNAs regulate multiple targets by recognizing a specific mRNA sequence in target mRNA. In T cells, miRNAs are involved in most processes and have important roles in T-cell activation and differentiation.^14^ A number of studies have profiled miRNAs in MS, and several miRNAs are now emerging as important regulators, one such being miR-21.^15^ Expression of miR-21 in CD4+ T cells has been shown to negatively correlate with DNA methylation at the CpGs in the *MIR21* gene.^16^

In this study, we sought to investigate DNA methylation in CD4+ T cells isolated from peripheral blood of RR-MS patients in remission, SP-MS patients, and healthy controls (HC).

## Materials and methods

Full details of experimental procedures are provided in Supplementary Methods.

### Cohorts

A discovery MS cohort and an independent cohort used for validation were recruited at the Neurology clinic at Karolinska University Hospital in Stockholm. Cohort details are provided in Table 1 and in Supplementary Table 1. The Regional Ethical Review Board in Stockholm approved this study (2009/2107-31/2 and 2010/879-31/1), and methods were carried out in accordance with institutional guidelines on human subject experiments. Informed consent was obtained from all subjects.

**Table 1.** Patient demographics in the discovery cohort used for 450K methylation analysis.

Status	Gender	Age	EDSS	MSSS	OCB	MRI lesions	TAS	TPS
RR-MS	F	37	3	2.91	Yes	10–20	Never Tx	
RR-MS	F	40	0	0.67	Yes	>20	Never Tx	
RR-MS	M	44	1.5		Yes	>20	Never Tx	
RR-MS	F	26	1	5.87	No	9	Never Tx	
RR-MS	F	29	2	5.87	Yes	>20	Never Tx	
RR-MS	F	35	1	1.13	Yes	>20	No Tx	Fingolimod
RR-MS	F	46	5	3.44	Yes	>20	IVIg	
RR-MS	F	37	1.5	3.34	Yes	>20	SD	
RR-MS	M	29	1	0.88	Yes	>20	No Tx	IFN
RR-MS	M	32	3	3.05	Yes	>20	No Tx	IFN
RR-MS	F	57	2.5	4.13	Yes	>20	No Tx	SD
RR-MS	F	41	2	0.71	Yes	>20	Never tx	
SP-MS	M	45	4	2.82	Yes	>20	No Tx	IFN
SP-MS	M	50	6.5	5.99	Yes	9	No Tx	SD
SP-MS	M	56	6.5	5.99	Yes	10–20	Never Tx	
SP-MS	F	63	5		Yes	>20	Never Tx	
SP-MS	F	35	5	7.32	Yes	>20	No Tx	IFN, GA
SP-MS	F	60	5	5.82	Yes	>20	No Tx	IFN, Mtx
SP-MS	M	44	6	5.43	Yes	>20	No Tx	IFN, SD
SP-MS	F	50	3.5	4.55	Yes	>20	No Tx	IFN
Summarized								
RR-MS	75%^a^	38 (26–57)^b^	2.0 (0–5.0)^b^	2.9 (0.7–5.9)^b^	92%^c^		17%^d^	
SP-MS	50%^a^	50 (35–63)^b^	5.2 (3.5–6.5)^b^	5.4 (2.8–7.3)^b^	100%^c^		0%^d^	
HC	67%^a^	42 (28–62)^b^	N/A	N/A	N/A		N/A	

RR: relapsing-remitting multiple sclerosis; SP: secondary-progressive multiple sclerosis; HC: healthy controls; F: female; M: male; EDSS: Expanded Disability Status Scale; MSSS: Multiple Sclerosis Severity Score; OCB: oligoclonal bands; MRI: magnetic resonance imaging; TAS: treatment at sampling; TPS: treatment prior to sampling; Tx: treatment; Never Tx: treatment naïve; No Tx: sampling after 6-month wash-out period; SD: study drug; IFN = interferon beta; GA: glatiramer acetate; Mtx: mitotrexate; N/A: not applicable; IVIg: intravenous Immunoglobulins.

a Percentage of females.

b Mean (range).

c Percentage of OCB-positive patients.

d Percentage of patients treated at the time of sampling.

The Genetics of Lipid Lowering Drugs and Diet Network (GOLDN) cohort recruited families with at least two siblings from the National Heart, Lung, and Blood Institute Family Heart Study sites at Minneapolis and Salt Lake City.^17–19^ The Institutional Review Board (IRB) approved the GOLDN study (E160405007).

### Preparation of CD4+ T cells

For discovery and validation cohorts, peripheral blood mononuclear cells (PBMCs) were isolated using a standard Ficoll (GE Healthcare) and sodium citrate–containing preparation tubes (Becton Dickinson) procedures, respectively. Sorting of the CD4^+^ T cells was performed on a MoFlo^™^ cell sorter (Beckman Coulter) and an autoMACS® cell separator (Miltenyi Biotec), respectively. Extraction of genomic DNA was carried out using GenElute Mammalian Genomic DNA Miniprep kit (Sigma-Aldrich). RNA was isolated using standard TRIzol protocol (Invitrogen) and Allprep Total RNA/DNA Kit (Qiagen), respectively.

### DNA methylation analysis

DNA methylation was profiled using the Infinium HumanMethylation450 BeadChip (Illumina; referred as 450K hereinafter) arrays at the Bioinformatics and Expression Analysis (BEA) core facility at Karolinska Institutet. Methylation data were individually analyzed using the minfi and ChAMP package and normalized using quantile normalization and Beta Mixture Quantile dilation (BMIQ). Differentially methylated positions were determined using the limma package applying a linear modeling that included MS status, age, and sex as covariates. A representative CpG was validated using pyrosequencing.

### Transcriptome analysis

Total RNA was subjected to the Illumina TruSeq Stranded mRNA Library Protocol with Dual Indexes (Illumina) and sequenced on Illumina HiSeq 2500 to generate 75 bp paired-end data with an average of 10 M reads above Q30. Differential expression analysis was conducted using the limma package.

Expression of miR-21 was determined using TaqMan MicroRNA Assay Kit (No.: 000397, Applied Biosystems), and *VMP1* expression was assessed by quantitative polymerase chain reaction (qPCR) using SYBR green chemistry (Bio-Rad).

### Genotyping

Allelic discrimination was performed using a predesigned TaqMan SNP Genotyping Assay (No.: 4351379, Applied Biosystems).

### Association, correlation, and meta-analysis

In the GOLDN cohort, we used the *lmekin* function of the R *kinship* package to fit linear mixed-effects models with the methylation β-value at each of the interrogated CpG sites as the outcome and different predictors.

In the MS cohort, we fit linear regression model in Rcmdr with the methylation β-value at each of the interrogated CpG sites as the outcome and different predictors.

We used two different meta-analysis methodologies: (1) the “summation of *p* value” method for combining *p*-values, and (2) the effect-size-based meta-analysis using a fixed-effects model (estimated by restricted maximum likelihood) since there was no evidence of heterogeneity.

### Target gene enrichment analysis

Targets of miR-21 identified in Jurkat T cells by RIP-Chip and predicted by TarBase7.0 were selected for analysis. Deviation of up-regulated and down-regulated miR-21 target genes from the expected ratio was calculated using the chi-square test. Enrichment of differentially expressed targets among up-regulated and down-regulated genes was calculated using Fisher’s exact test.

### Ingenuity pathway analysis

Upstream regulators and biological functions were determined in the ingenuity pathway analysis (IPA) platform (Qiagen) using Fisher’s exact test and right-tailed Fisher’s exact test, followed by Benjamini–Hochberg correction, respectively.

## Results

### CD4+ T cells from RR-MS patients display higher DNA methylation in the *VMP1/MIR21* locus

**Figure 1.**
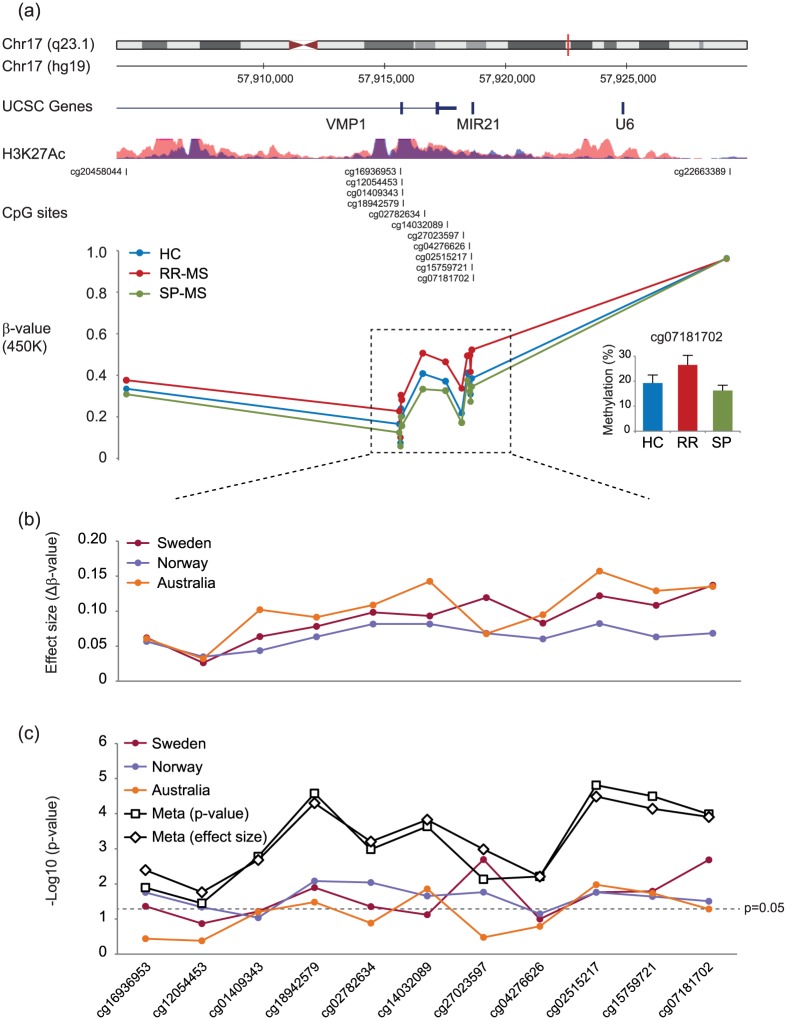
CD4+ T cells from RR-MS patients display higher DNA methylation levels in the *VMP1/MIR21* locus. (a) DNA methylation was measured using 450K arrays in CD4+ T cells sorted from peripheral blood of RR-MS (*n* = 12) and SP-MS (*n* = 8) patients and healthy controls (HC; *n* = 12). Eleven consecutive CpG sites (dashed box) displayed significantly higher methylation in RR-MS compared to SP-MS and HC. Detailed analysis of the locus is given in Table 2. The CpGs map to the *VMP1* gene (last two exons and intron) and *MIR21* gene, the region that is enriched for active histone mark (H3K27Ac) in two immune cell lines (ENCODE: GM12878 and K562). The methylation difference at a selected CpG, cg07181702 in the *MIR21* gene, was technically validated using pyrosequencing (right lower panel). (b) The effect size that represents a difference in β-values between RR-MS and HC at a given CpG displayed the same pattern of increased DNA methylation in RR-MS compared to HC at the 11 consecutive CpG sites in the Swedish (*N* = 24) cohort and two additional cohorts from Australia (*N* = 40)^11^ and Norway (*N* = 30).^12^ (c) Comparison of RR-MS with HC was performed on β-values and corrected for age in the Norwegian and Australian cohort (comprising females only), and age and sex in the Swedish cohort. Meta-analysis performed combining *p*-values (using the “summation of *p* value” method) and effect sizes (using fixed-effects model) yielded significant *p*-values for all 11 CpGs. Significance levels for each CpG in different analyses are given on the *y*-axis as a −log10(*p*-value).

We investigated DNA methylation in CD4+ T cells from a Swedish cohort comprising RR-MS, SP-MS, and matched HC. Analysis of 432,874 probes from the 450K did not yield genome-wide significant changes. However, among the top most significant hits, we observed multiple probes mapping to last two exons of *VMP1* and *MIR21* genes (Figure 1(a); Table 2). RR-MS displayed significantly higher methylation compared to HC and SP-MS at the 11 consecutive CpG probes. The cg07181702 in *MIR21*, which was validated using pyrosequencing (Figure 1(a)), had the largest effect size of 14% methylation change between RR-MS and HC.

**Table 2.** DNA methylation changes in the *VMP1/MIR21* locus in CD4+ T cells from RR-MS and SP-MS patients and HC.

Probe ID^a^	Gene^a^	Feature^a^	ALL^b^	RR vs HC^b^		RR vs SP^b^		SP vs HC^b^	
			*p*-val (*M*)	Eff (Δβ)	*p*-val (*M*)	Eff (Δβ)	*p*-val (*M*)	Eff (Δβ)	*p*-val (*M*)
cg20458044	VMP1	Intron	1E-01	0.04	2E-01	0.07	**4E-02**	–0.03	3E-01
**cg16936953**	VMP1	Exon	**2E-03**	0.06	6E-02	0.10	**5E-04**	–0.04	4E-02
**cg12054453**	VMP1	Exon	**4E-03**	0.03	9E-02	0.04	**1E-03**	–0.02	5E-02
**cg01409343**	VMP1	Exon	**1E-02**	0.06	7E-02	0.10	**3E-03**	–0.04	2E-01
**cg18942579**	VMP1	Intron	**4E-04**	0.08	**1E-02**	0.12	**1E-04**	–0.05	6E-02
**cg02782634**	VMP1	Intron	**4E-03**	0.10	**5E-02**	0.17	**1E-03**	–0.08	8E-02
**cg14032089**	MIR21	TSS1500	**3E-02**	0.09	7E-02	0.14	**1E-02**	–0.05	3E-01
**cg27023597**	MIR21	TSS1500	**2E-04**	0.12	**2E-03**	0.16	**6E-05**	–0.04	1E-01
**cg04276626**	MIR21	TSS200	7E-02	0.08	1E-01	0.12	**3E-02**	–0.04	4E-01
**cg02515217**	MIR21	TSS200	**4E-03**	0.12	**2E-02**	0.17	**2E-03**	–0.05	3E-01
**cg15759721**	MIR21	Body	**9E-03**	0.11	**4E-02**	0.14	**3E-03**	–0.04	2E-01
**cg07181702**	MIR21	Body	**2E-03**	0.14	**7E-03**	0.18	**9E-04**	–0.04	3E-01
cg22663389	–	IGR	6E-01	–0.00	3E-01	–0.00	6E-01	–0.00	7E-01

MS: multiple sclerosis; RR: relapsing-remitting multiple sclerosis; SP: secondary-progressive multiple sclerosis; HC: healthy controls.

a CpG probe ID, annotated gene names and genomic features from 450K arrays.

b *M*-values (indicated by *M*) were used to compare RR-MS patients (RR; *n* = 12), SP-MS patients (SP; *n* = 8), and healthy controls (HC; *n* = 12), and *p*-values (*p*-val) were derived using a linear modeling that included MS status, age, and sex as covariates. The effect size (Eff) represents a difference in β-values (Δβ) between the groups for each probe. *p*-val <0.05 are indicated in bold.

We sought to replicate our findings between RR-MS and HC (*n* = 24) in independent CD4+ T-cell cohorts comprising RR-MS from Australia (*N* = 40) and Norway (*N* = 30).^11,12^ Higher methylation in RR-MS was observed at all 11 CpGs in both cohorts (Figure 1(b)). Of the 11 CpGs, 9 and 4 displayed significant changes in the Norwegian and Australian cohort, respectively (Figure 1(c)). Meta-analysis performed combining *p*-values and effect sizes from all three cohorts demonstrated significant changes for all 11 CpGs (Figure 1(c)).

These data support higher methylation levels in the *VMP1/MIR21* locus in RR-MS patients in three independent cohorts.

### Investigation of association between methylation levels in *VMP1/MIR21* and MS risk factors

**Figure 2.**
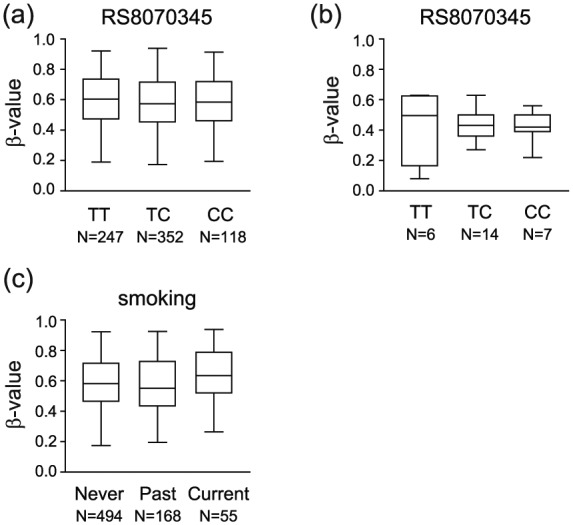
MS risk factors, genetic variation in the *VMP1* gene and smoking, do not affect DNA methylation in the *VMP1/MIR21* locus. DNA methylation levels (β-values) in the *VMP1/MIR21* locus, exemplified by cg07181702, do not associate with the MS risk genotype at RS8070345 (T = risk allele) in a (a) large cohort of healthy individuals or (b) in the Swedish cohort. Detailed analysis of the locus is given in Tables 3 and 4. (c) DNA methylation levels (β-values) in the *VMP1/MIR21* locus, exemplified by cg07181702, are not affected by the smoking status in a large cohort of healthy individuals. Detailed analysis of the locus is given in Table 3.

It has been shown that genetic variants can mediate disease risk through changes in DNA methylation,^20^ and there is a well-established genetic association between MS and the *VMP1* locus.^4^ Thus, we investigated whether genetic variation in *VMP1* may mediate MS risk by affecting *VMP1/MIR21* methylation levels. We detected significant differences in CD4+ T cells from healthy individuals in the GOLDN cohort (*N* = 717)^17–19^ for only 2 of 11 CpGs comparing individuals carrying none, one, or two of the RS8070345 risk alleles (Figure 2(a); Table 3) and no significant differences in the MS cohort (Figure 2(b); Table 4).

**Table 3.** Influence of RS8070345, smoking, and age on DNA methylation in the *VMP1/MIR21* locus in healthy individuals.

CpG probe^a^	Gene^a^	RS8070345^b^ (*N* = 717)		Current smoker^c^ (*N* = 994)		Past smoker^c^ (*N* = 994)		Age^c^ (*N* = 994)	
		Coeff	*p*-val	Coeff	*p*-val	Coeff	*p*-val	Coeff	*p*-val
cg16936953	VMP1	–0.0106	**0.049**	–0.0098	0.34	–0.0060	0.37	–0.0010	**5E-07**
cg12054453	VMP1	–0.0170	**1E-04**	–0.0158	0.07	–0.0120	**0.031**	–0.0005	**4E-03**
cg01409343	VMP1	–0.0046	0.43	0.0011	0.92	0.0002	0.97	–0.0011	**2E-07**
cg18942579	VMP1	–0.0086	0.13	0.0000	1.00	0.0020	0.78	–0.0011	**3E-07**
cg02782634	VMP1	0.0016	0.78	–0.0065	0.55	0.0007	0.92	–0.0010	**5E-07**
cg14032089	MIR21	0.0057	0.33	–0.0041	0.71	0.0019	0.79	–0.0009	**2E-05**
cg27023597	MIR21	–0.0058	0.34	–0.0126	0.28	0.0002	0.98	–0.0010	**5E-06**
cg04276626	MIR21	0.0032	0.58	–0.0057	0.59	0.0028	0.68	–0.0004	0.06
cg02515217	MIR21	0.0016	0.82	0.0022	0.85	–0.0001	0.99	–0.0012	**3E-07**
cg15759721	MIR21	0.0048	0.42	–0.0017	0.88	0.0019	0.79	–0.0010	**1E-06**
cg07181702	MIR21	0.0034	0.57	0.0011	0.92	0.0014	0.85	–0.0011	**4E-07**

a CpG probe ID and annotated gene names from 450K arrays.

b Coefficients (Coeff) and *p*-values (*p*-val) from a linear mixed-effects model with the methylation β-score at each CpG site as the outcome and the following predictors: genotype at RS8070345, age, sex, study site, current smoking, body mass index (BMI), principal components capturing T-cell purity, and family, as previously described.^19^ *p*-val <0.05 are indicated in bold.

c Coefficients (Coeff) and *p*-values (*p*-val) from a linear regression model with the methylation β-score at each CpG site as the outcome and the following predictors: age, current and past smoking, BMI, and principal components capturing T-cell purity, as previously described.^19^ *p*-val <0.05 are indicated in bold.

**Table 4.** Correlation of DNA methylation in the *VMP1/MIR21* locus with patient characteristics.

CpG probe^a^	Gene^a^	RS8070345^b^ (*N* = 27)		Ly#^c^ (*N* = 18)		Age^c^ (*N* = 18)	
		Coeff	*p*-val	Coeff	*p*-val	Coeff	*p*-val
cg16936953	VMP1	–0.0076	0.59	0.0534	0.10	–0.0067	**5E-05**
cg12054453	VMP1	–0.0061	0.36	0.0178	0.37	–0.0027	**3E-03**
cg01409343	VMP1	0.0003	0.98	0.0788	0.078	–0.0070	**2E-04**
cg18942579	VMP1	–0.0013	0.94	0.0510	0.22	–0.0055	**6E-03**
cg02782634	VMP1	–0.0275	0.32	0.1077	0.12	–0.0096	**2E-03**
cg14032089	MIR21	–0.0111	0.69	0.0956	0.16	–0.0101	**1E-03**
cg27023597	MIR21	0.0090	0.67	0.1034	**0.039**	–0.0077	**5E-04**
cg04276626	MIR21	–0.0104	0.72	0.0955	0.15	–0.0103	**8E-04**
cg02515217	MIR21	–0.0162	0.56	0.0582	0.38	–0.0112	**4E-04**
cg15759721	MIR21	–0.0134	0.61	0.1116	0.082	–0.0086	**2E-03**
cg07181702	MIR21	–0.0053	0.84	0.0664	0.31	–0.0105	**7E-04**

a CpG probe ID and annotated gene names from 450K arrays.

b Coefficients (Coeff) and *p*-values (*p*-val) from a linear regression model with the methylation β-score at each CpG site as the outcome and the following predictors: age, sex, and genotype at RS8070345 (*N* = 27).

c Coefficients (Coeff) and *p*-values (*p*-val) from a linear regression model with the methylation β-score at each CpG site as the outcome and the following predictors: age, sex, and lymphocyte count (Ly#) (*N* = 18). *p*-val <0.05 are indicated in bold.

We then tested whether *VMP1/MIR21* methylation levels mediate the effect of smoking, a well-established risk factor for MS^5^ also known to affect DNA methylation.^21^ No consistent significant differences at any of the 11 CpGs between never, past, and current smokers were detected in the GOLDN cohort (Figure 2(c); Table 3).

**Figure 3.**
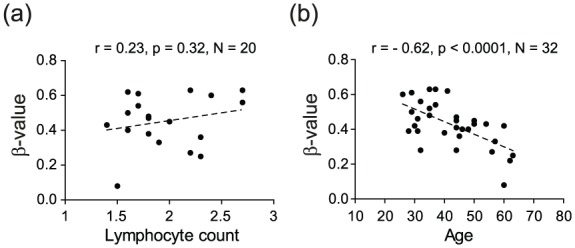
DNA methylation in the *VMP1/MIR21* locus displays strong negative correlation with age. DNA methylation levels (β-values) in the *VMP1/MIR21* locus, exemplified by cg07181702, do not show significant association with (a) lymphocyte count, as a surrogate of inflammation levels, but display a significant strong negative correlation with (b) age. Detailed analysis of the locus is given in Table 4.

There was a tendency for a positive correlation with lymphocyte counts with 1/11 and 3/11 CpGs displaying *p* < 0.05 and *p* < 0.1, respectively (Figure 3(a); Table 4). Additionally, methylation in *VMP1/MIR21* displayed a strong negative correlation with age (e.g. for cg07181702, *n* = 32, Pearson’s *r* = –0.62, *p* < 0.0001) (Figure 3(b); Table 4), with higher methylation levels in CD4+ T cells of younger individuals, which was also detected in the GOLDN cohort (e.g. for cg07181702, *n* = 994, coefficient = –0.0011, *p* < 4 × 10^−7^) (Table 3).

Thus, increased methylation in *VMP1/MIR21* is not caused by the genetic variation in the locus or smoking, but instead age strongly affects *VMP1/MIR21* methylation.

### Hypermethylation of the *VMP1/MIR21* locus associates with lower expression of miR-21 in CD4+ T cells

**Figure 4.**
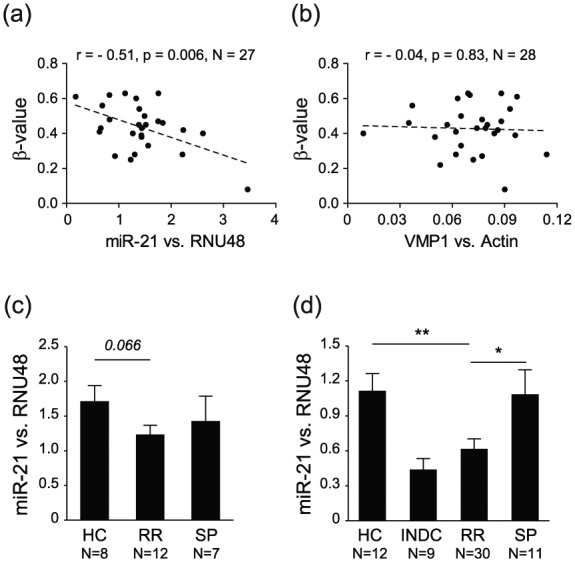
DNA methylation in the *VMP1/MIR21* locus displays significant negative correlation with miR-21 expression, and RR-MS patients display lower miR-21 levels. DNA methylation levels (β-values) in the *VMP1/MIR21* locus, exemplified by cg07181702, display a significant strong anti-correlation with (a) miR-21 levels but not with (b) *VMP1* levels in CD4+ T cells from RR-MS, SP-MS and healthy controls (HC). Accordingly, RR-MS patients have lower levels of miR-21 compared to HC and SP-MS patients in the (c) 450K cohort and in an (d) independent validation cohort from Sweden. Detailed analysis of the locus is given in Table 5.

There was a high correlation between methylation levels at all CpGs (Supplementary Figure 1), suggesting that methylation changes in the region are co-regulated and likely have the same functional impact. We then investigated whether methylation in *VMP1/MIR21* has an impact on the expression of miR-21 and *VMP1*, analyzed by qPCR in patients and HC from the MS cohort. We observed a significant negative correlation between methylation at 7/11 CpGs and miR-21 levels, with additional 4/11 CpGs displaying the same trend (Figure 4(a); Table 5), while there was no correlation with *VMP1* levels (Figure 4(b); Table 5). This inverse correlation between methylation and miR-21 levels was strongest for the CpGs in *MIR21* (e.g. for cg 07181702, *n* = 27, Pearson’s *r* = –0.51, *p* = 0.006) and remained significant after age correction (Table 5).

**Table 5.** Correlation of DNA methylation in the *VMP1/MIR21* locus with miR-21 and *VMP1* expression levels.

CpG probe^a^	Gene^a^	miR-21^b^ (*N* = 27)				*VMP1*^b^ (*N* = 27)	
		*r*	*p*-val	Coeff	*p*-val	*r*	*p*-val
cg16936953	VMP1	–0.34	0.083	–3.49	0.14	–0.02	0.91
cg12054453	VMP1	–0.23	0.24	–3.84	0.42	0.02	0.90
cg01409343	VMP1	–0.40	**0.038**	–3.85	0.054	–0.06	0.77
cg18942579	VMP1	–0.32	0.108	–2.53	0.21	0.09	0.66
cg02782634	VMP1	–0.42	**0.027**	–2.39	**0.042**	–0.11	0.57
cg14032089	MIR21	–0.38	**0.047**	–2.11	0.081	–0.08	0.70
cg27023597	MIR21	–0.35	0.076	–2.50	0.12	0.13	0.52
cg04276626	MIR21	–0.54	**0.004**	–3.08	**0.006**	–0.04	0.82
cg02515217	MIR21	–0.43	**0.025**	–2.82	**0.029**	0.08	0.68
cg15759721	MIR21	–0.50	**0.008**	–3.20	**0.013**	–0.06	0.76
cg07181702	MIR21	–0.51	**0.006**	–3.32	**0.006**	–0.04	0.83

RR: relapsing-remitting multiple sclerosis; SP: secondary-progressive multiple sclerosis; HC: healthy controls.

a CpG probe ID and annotated gene names from 450K arrays.

b Pearson’s *r* and regression coefficients (Coeff), with accompanying *p*-values (*p*-val), from a Pearson correlation test, for relative expression of miR-21 and *VMP1*, and a linear regression model with the relative expression of miR-21 as the outcome and methylation β-score at each CpG site and age as predictors. *p*-val < 0.05 are indicated in bold. Analysis was performed in CD4+ T cells from RR-MS, SP-MS, and HC.

Accordingly, a tendency for decrease in the expression of miR-21 was observed in RR-MS patients compared to SP-MS and HC (Figure 4(c)). To validate our findings, we performed expression analysis in an independent MS cohort from Sweden including more cases (Supplementary Table 1). Significantly lower levels of miR-21 were detected in RR-MS patients compared to SP-MS patients and HC (Figure 4(d)).

These data strongly suggest that increased methylation in *MIR21* associates with lower expression of mature miR-21 in CD4+ T cells of RR-MS patients.

### MiR-21 target genes are enriched among genes up-regulated in CD4+ T cells of RR-MS patients

Since the best described function of miRNAs is to induce degradation of target mRNAs, we explored whether the observed changes in miR-21 methylation and expression lead to actual functional changes in miR-21 target genes. For that purpose, we used RNA-seq data from a cohort largely overlapping (73%) the cohort used for 450K analysis (Supplementary Table 1). We used miR-21 targets identified in Jurkat T cells using RIP-Chip and predicted by TarBase7.0 (Supplementary Table 2). We first investigated whether the ratio of up-regulated miR-21 targets deviated from the expected ratio of up-regulated genes between RR-MS, SP-MS, and HC (Table 6). There was a significant enrichment of miR-21 targets among the genes that were up-regulated in CD4+ T cells, both when comparing RR-MS patients with HC and when comparing RR-MS with SP-MS but not when SP-MS patients were compared with HC (Table 6). Similar results were obtained when we calculated enrichment of miR-21 targets among up-regulated and down-regulated genes where we found significant enrichment of miR-21 targets predominantly among up-regulated, but not down-regulated, genes between RR-MS and HC (Table 7). Additionally, an unbiased IPA prediction of upstream regulators identified miR-21 as a significantly inhibited upstream regulator (*z* = –2.1, *p* = 7 × 10^−7^) in RR-MS that can explain the observed pattern of differential gene expression between RR-MS and HC (*p* < 0.05).

**Table 6.** Deviation of the miR-21 target genes from the expected ratio of the up-regulated genes in CD4+ T cells from RR-MS and SP-MS patients and HC.

Prediction^a^	Comparison^a^	DE^b^ (*p*-val)	Group^b^	UP obs^b^ (%)	UP exp^b^ (%)	*p*-val^b^
Jurkat (*N* = 64)	RR vs HC	0.01	RR	100	53	0.1
		0.05	RR	80	44	**0.02**
		0.1	RR	86	43	**0.001**
	RR vs SP	0.01	RR	–	35	
		0.05	RR	100	34	**0.006**
		0.1	RR	78	37	**0.01**
	SP vs HC	0.01	SP	100	63	0.4
		0.05	SP	100	58	0.1
		0.1	SP	78	56	0.2
TarBase (*N* = 433)	RR vs HC	0.01	RR	90	53	**0.02**
		0.05	RR	65	44	**0.004**
		0.1	RR	61	43	**0.002**
	RR vs SP	0.01	RR	75	35	0.09
		0.05	RR	64	34	**0.004**
		0.1	RR	68	37	**0.00001**
	SP vs HC	0.01	SP	50	63	0.5
		0.05	SP	52	58	0.5
		0.1	SP	52	56	0.6

RR: relapsing-remitting multiple sclerosis; SP: secondary-progressive multiple sclerosis; HC: healthy controls.

a Target genes of miR-21 identified in Jurkat T cells (*N* = 64, 100% present in RNA-seq data) and genes predicted and experimentally validated to be miR-21 targets by TarBase7.0 (*N* = 433, 88% present in RNA-seq data) were selected for analysis.

b Differentially expressed (DE) genes between RR-MS patients (RR; *n* = 12), SP-MS patients (SP; *n* = 10), and healthy controls (HC; n = 12) were identified using RNA-seq (significance for calling DE is indicated by DE (*p*-val)). For each comparison, a deviation of the percentage of the observed up-regulated miR-21 target genes (UP obs), in a given group (Group), from the expected percentage of the up-regulated genes (UP exp) was calculated using a chi-square test (*p*-val). *p*-val <0.05 are indicated in bold.

**Table 7.** Overlap of miR-21 target genes with up-regulated and down-regulated genes in CD4+ T cells from RR-MS and SP-MS patients and HC.

Prediction^a^	Comparison^a^	DE (*p*-val)^b^	*p*-val (UP)^b^	*p*-val (DOWN)^b^
Jurkat (*N* = 64)	RR vs HC	0.01	***0.06***	0.8
		0.05	**0.003**	0.6
		0.1	**0.0009**	0.1
	RR vs SP	0.01	-	-
		0.05	0.1	0.1
		0.1	***0.06***	0.2
	SP vs HC	0.01	0.9	0.7
		0.05	0.4	0.4
		0.1	0.3	0.6
TarBase (*N* = 433)	RR vs HC	0.01	***0.09***	0.2
		0.05	**0.0005**	0.4
		0.1	**0.003**	0.1
	RR vs SP	0.01	0.8	0.7
		0.05	0.2	**0.02**
		0.1	**0.003**	**0.002**
	SP vs HC	0.01	0.8	0.6
		0.05	0.9	0.3
		0.1	0.3	0.1

RR: relapsing-remitting multiple sclerosis; SP: secondary-progressive multiple sclerosis; HC: healthy controls.

a Target genes of miR-21 identified in Jurkat T cells (*N* = 64, 100% present in RNA-seq data) and genes predicted and experimentally validated to be miR-21 targets by TarBase7.0 (*N* = 433, 88% present in RNA-seq data) were selected for analysis.

b Differentially expressed (DE) genes between RR-MS patients (RR; *n* = 12), SP-MS patients (SP; *n* = 10), and healthy controls (HC; *n* = 12) were identified using RNA-seq (significance for calling DE is indicated by DE (*p*-val)). For each comparison, an overlap of miR-21 target genes with either up-regulated (UP) or down-regulated (DOWN) genes was calculated using Fisher’s exact test (*p*-val). *p*-val <0.05 and <0.1 are indicated in bold and italic bold, respectively.

In order to identify biological functions that are regulated by miR-21 in CD4+ T cells we performed IPA on TarBase7.0-predicted miR-21 targets that were up-regulated in RR-MS compared to HC (p<0.05, n=30) (Supplementary Table 2). Despite a limited list of genes several functions were significant after Benjamini-Hochberg correction including “*Apoptosis*” (z=-1.624) and “*Proliferation of cells*” (z=1.007).

These analyses strongly suggest a link between lower miR-21 expression and up-regulation of a set of miR-21 target genes in CD4+ T cells that potentially influence apoptosis and cell proliferation.

## Discussion

We performed DNA methylation analysis in CD4+ T cells from RR-MS, SP-MS, and healthy individuals and subsequently characterized the relationship between *MIR21* methylation and expression of miR-21 and its target genes. *MIR21* displayed increased methylation levels that correlated with a lower expression of mature miR-21 and an enrichment of up-regulated miR-21 target genes in RR-MS patients.

In order to assess the relevance of *MIR21* hypermethylation in RR-MS, we investigated both the methylation of this locus in previously published MS cohorts and assessed potential functional impact of *MIR21* methylation using RNA-seq data. A similar pattern as in our study, that is, hypermethylation of all 11 CpGs in RR-MS, was observed in the Norwegian and Australian cohorts,^11,12^ with nine and four significant probes (*p* < 0.05), respectively (two additional probes from each cohort demonstrated *p* < 0.1). The most significant changes were observed in the Norwegian cohort, which included untreated MS patients with relatively benign disease course^12^ that can potentially explain a more modest effect size of 6%–8% change in *MIR21*. The effect size was similar in the Swedish and Australian cohorts ranging from 8% to 14% and 7% to 16%, respectively, but the changes were less significant in the Australian cohort potentially due to higher variability caused by treatments.^11^ It is important to consider that all studies investigated bulk CD4+ T cells that comprise subsets with distinct methylomes,^16,22^ and methylation changes could reflect differences in their cellular composition. However, as *MIR21* is hypomethylated in Th1/Th2^22^ and regulatory T cells (Tregs)^16^ compared to naïve CD4+ T cells, and naive CD4+ T cells display the lowest expression of miR-21 compared to other CD4+ subsets,^14^ the differences in frequency are less likely to explain hypermethylation in RR-MS.

We further demonstrated a functional impact of the observed methylation on the expression of miR-21. The higher methylation levels in *VMP1/MIR21* correlated strongly with lower expression of mature miR-21 in CD4+ T cells, whereas no correlation was observed with *VMP1*. The negative correlation with miR-21 levels was the strongest at the CpGs in the *MIR21* gene. Methylation at the same CpGs has previously been shown to negatively correlate with miR-21 expression in naïve CD4+ T cells upon activation.^16^ Accordingly, we observed lower expression of mature miR-21 in RR-MS patients compared to SP-MS and controls, and this difference became significant in a larger independent cohort. The difference between RR-MS and SP-MS, however, may reflect differences in age between the groups since *MIR21* methylation displays a strong negative correlation with age.

Consistent with the function of miRNAs in down-regulating their target genes, we found miR-21 targets enriched among up-regulated genes in RR-MS patients. The enrichment was significant irrespective of the target prediction tool, the cut-off levels for differential expression, and the enrichment analysis approach used. Importantly, the enrichment was detected only in RR-MS, which is in line with the observed higher methylation and lower expression of miR-21 in RR-MS compared to HC or SP-MS and no difference between HC and SP-MS. The most prominent biological functions that associated with the up-regulated targets imply possible anti-apoptotic and pro-proliferative effects. Representatives for such genes are *DEGS1, PTPN13*, and *CD47* that have been shown to inhibit apoptosis and increase proliferation in different cells. For instance, DEGS1, enzyme involved in the ceramide synthesis, has been shown to increase proliferation and decrease apoptosis in mammalian cells.^23^ In CD4+ T cells, ceramides have been implicated in regulating T-cell proliferation and differentiation into Th1.^24^ Overexpression of PTPN13, a protein tyrosine phosphatase, leads to increased resistance to FAS-induced apoptosis in immortalized T cells.^25^ The ligation of CD47, a plasma membrane protein, leads to an increase in interleukin (IL)-2 production and more proliferation in primary and Jurkat T cells.^26^ Gene deletion or blocking of CD47 protects against development of MS-like disease in mice through a failure of immune cell activation.^27^ In relation to these observations, our data suggest a potential role of methylation-dependent miR-21 expression in modulating proliferation and apoptosis of CD4+ T cells in RR-MS.

Expression of miR-21 is increased in many inflammatory diseases, and elevated miR-21 levels represent a sign of inflammation.^16,28^ In MS, miR-21 has been found to be up-regulated in PBMCs of RR-MS patients in relapse, suggesting that hypermethylation of *MIR21* may be a mechanism to down-regulate miR-21 that may contribute to remission.^29^ However, another longitudinal study did not report differences in miR-21 expression in peripheral blood leukocytes from RR-MS patients in relapse and remission, but did observe down-regulation of miR-21 in RR-MS patients in remission compared to controls,^30^ which is similar to our observation. Regarding T cells, miR-21 has been shown to both promote and inhibit T-cell activation.^31,32^ Overexpression of miR-21 in naïve mouse T cells promotes expression of Th2 and Treg genes^33^ and expression of FoxP3 in human Tregs.^34^ Accordingly, lower expression of miR-21 has been reported in PBMCs and CD4+ T cells of rheumatoid arthritis patients compared to controls, with low miR-21 showing strong correlation with an increased ratio of Th17/Treg cells.^35^ Arguably, miR-21 may play different roles in different stages of disease depending on the stage of CD4+ T-cell activation and the cell subset.

We also investigated potential causes of the observed changes in DNA methylation. Since *MIR21* resides in the locus that associates with susceptibility to MS,^4^ we first investigated whether methylation of *MIR21* is genetically regulated. However, we found no consistent evidence of association of the risk variant with the levels of *MIR21* methylation. Similarly, we found no influence of smoking, which is known to induce DNA methylation changes^21^ and is an established lifestyle MS risk factor.^5^ One possibility is that *MIR21* methylation and expression are regulated by the inflammation itself. The fact that miR-21 expression does not differ in SP-MS but shows low expression in RR-MS and inflammatory neurological disease controls supports this scenario. This is further supported by the observed trend for positive correlation between methylation and lymphocyte counts, as a surrogate of inflammation. However, none of the known proinflammatory regulators of miR-21^28^ were differentially expressed between RR-MS and SP-MS patients or HC, suggesting that maybe differential methylation in their binding sites in *MIR21* may lead to differences in miR-21 expression. Whether this is the case and the exact mechanisms of how inflammatory signals induce epigenetic repression in the locus need to be established.

To our knowledge, this is the first study that delivers a detailed description of the methylation status of *MIR21* in CD4+ T cells of MS patients and associates it with the nearby risk allele, smoking, age, and changes in expression of miR-21 and its target genes. Our study sheds further light on the role of miR-21 in inflammatory diseases, in particular MS, demonstrating that disease-related changes in an epigenetic marking have the potential to lead to differences in the expression of miR-21 with a consequence on miR-21 target genes.

## Supplementary Material

Supplementary material

Supplementary material

Supplementary material

Supplementary material
