# Supplementary material for: Hypermethylation of MIR21 in CD4+ T cells from patients with relapsing-remitting multiple sclerosis associates with lower miRNA-21 levels and concomitant up-regulation of its target genes
Source: Mult Scler. 2017 Aug 2;24(10):1288–300. doi: 10.1177/1352458517721356 (PMC5794671; doi:10.1177/1352458517721356)
Supplement: Supplementary material [file MSJ721356_supplementary_figure_1.pdf]

Supplementary Figure 1

		cg16936953	cg12054453	cg01409343	cg18942579	cg02782634	cg14032089	cg27023597	cg04276626	cg02515217	cg15759721	cg07181702
cg16936953	VMP1 (Exon)	1,00	0,89	0,94	0,93	0,90	0,89	0,90	0,82	0,94	0,92	0,89
cg12054453	VMP1 (Exon)	0,89	1,00	0,88	0,87	0,74	0,73	0,82	0,67	0,82	0,76	0,75
cg01409343	VMP1 (Exon)	0,94	0,88	1,00	0,92	0,81	0,83	0,87	0,74	0,89	0,85	0,86
cg18942579	VMP1 (Intron)	0,93	0,87	0,92	1,00	0,83	0,79	0,89	0,72	0,87	0,85	0,83
cg02782634	VMP1 (Intron)	0,90	0,74	0,81	0,83	1,00	0,89	0,78	0,91	0,91	0,96	0,91
cg14032089	MIR21 (TSS1500)	0,89	0,73	0,83	0,79	0,89	1,00	0,81	0,87	0,91	0,91	0,87
cg27023597	MIR21 (TSS1500)	0,90	0,82	0,87	0,89	0,78	0,81	1,00	0,73	0,87	0,83	0,84
cg04276626	MIR21 (TSS200)	0,82	0,67	0,74	0,72	0,91	0,87	0,73	1,00	0,87	0,91	0,86
cg02515217	MIR21 (TSS200)	0,94	0,82	0,89	0,87	0,91	0,91	0,87	0,87	1,00	0,92	0,94
cg15759721	MIR21 (Body)	0,92	0,76	0,85	0,85	0,96	0,91	0,83	0,91	0,92	1,00	0,92
cg07181702	MIR21 (Body)	0,89	0,75	0,86	0,83	0,91	0,87	0,84	0,86	0,94	0,92	1,00

Pearson r
0,00
0,10
0,20
0,30
0,40
0,50
0,60
0,70
0,80
0,90
1,00

Supplementary Figure 1: Correlation between DNA methylation levels at 11 CpGs in the region. Analysis was performed on β-values from RR-MS, SP-MS and HC (n=32) using the Pearson correlation test and Pearson's r is displayed.
