# Supplementary material for: Hypermethylation of MIR21 in CD4+ T cells from patients with relapsing-remitting multiple sclerosis associates with lower miRNA-21 levels and concomitant up-regulation of its target genes
Source: Mult Scler. 2017 Aug 2;24(10):1288–300. doi: 10.1177/1352458517721356 (PMC5794671; doi:10.1177/1352458517721356)
Supplement: Supplementary material [file MSJ721356_supplementary_methods.pdf]

Supplementary Methods

Samples

Multiple Sclerosis cohorts

A discovery cohort comprised RR-MS, SP-MS and matched healthy controls (HC) recruited at the local Neurology clinic at the Karolinska University Hospital in Stockholm, Sweden. Detailed patient demographics are provided in Table 1. An independent cohort comprising RR-MS, SP-MS, inflammatory neurological disease controls (INDC) and HC was recruited at the same clinic and used for validation. Detailed patient demographics are provided in Table S1. All patients had given informed consent and the study was approved by the ethical review board of Stockholm (2009/2107-31/2 and 2010/879-31/1).

The GOLDN cohort

The Genetics of Lipid Lowering Drugs and Diet Network (GOLDN) study, aimed at investigating genetic and epigenetic determinants of response to interventions targeting blood lipid levels, recruited families with at least two siblings from the National Heart, Lung, and Blood Institute Family Heart Study sites at Minneapolis and Salt Lake City. The study obtained epigenome-wide DNA methylation data on CD4+ T-cells using the Illumina Infinium 450K Human Methylation Beadchip as described in previous publications[1-3]. Genotyping of GOLDN participants was performed using the Affymetrix Genome-Wide Human 6.0 array[4]. After all quality control procedures were performed, 717 out of 994 participants had both genetic and epigenetic data and were included in this analysis. The Institutional Review Board (IRB) approved the GOLDN study (E160405007).

Preparation of CD4+ T cells

For the discovery cohort peripheral blood mononuclear cells (PBMCs) were isolated and collected in sodium heparin tubes using a standard Ficoll (GE Healthcare) procedure. Cells were separated using density gradient centrifugation, collected from the interphase, washed twice in Dulbecco's phosphate buffered saline and prepared for cell sorting. Sorting of the $CD4^+$ T cell population was performed by adding fluorochrome-conjugated antibodies against human CD4 (Becton Dickinson) and CD3 (BD Bioscience) using a MoFlo high-speed cell sorter (Beckman Coulter).

For the validation cohort PBMCs were isolated from peripheral blood using sodium citrate-containing cell preparation tubes (BD Vacutainer™ CPT™ Tube, Becton Dickinson), Sorting of the CD4+ T cell population was performed by adding microbeads against human CD4 using an autoMACS® cell separator (Miltenyi Biotec).

Directly after sorting, cell pellets were frozen and kept at -80C° until DNA/RNA extraction.

DNA extraction

Extraction of genomic DNA was carried out using a Gen Elute Mammalian Genomic DNA Miniprep kit (Sigma-Aldrich). The amount and quality of DNA was accessed by NanoDrop ND-1000 Spectrophotometer (NanoDrop Technologies).

DNA methylation analysis

DNA methylation was profiled using the Infinium HumanMethylation450 BeadChip (Illumina) arrays at the bioinformatics and expression analysis core facility (BEA) at Karolinska Institutet. Methylation data was individually analyzed in R using the Minfi and ChAMP package, probes containing SNPs within 2 bp of the CpG were removed, and type 1 and type 2 probes were

normalized using quantile normalization and BMIQ[5]. The sex of the patients was confirmed using the GetSex function from the Minfi package and the cell type was confirmed using the cell type deconvolution method from Minfi based on the Houseman algorithm[6]. Significant cofounders were identified using PCA. Batch effects were corrected using ComBat from the SVA package[7]. Differentially methylated positions (DMPs) were determined by using the limma package[8] applying a linear modeling that included MS status, age and sex as co-variates. Differences were calculated between HC and RR-MS and RR-MS and SP-MS. Additionally any significant differences between all three groups were identified using the moderated F-statistic test included in eBayes[9].

Meta-analysis

Meta-analysis on the 11 CpG probes was performed by combining the outcomes of the analyses between RR-MS and HC using β-values from the cohorts from Sweden (n=24), Norway (n=30) and Australia (n=40). Comparison of RR-MS with HC was performed on β-values and corrected for age in the Norwegian and Australian cohort (comprising females only), and age and sex in the Swedish cohort. Due to the strong impact of age on the methylation in the *MIR21* region only individuals with available age information and age-matched HC where retained for analysis. Similar data were obtained with all individuals (data not shown). We used two different meta-analysis methodologies: (1) the "summation of p value" method for combining p-values[10], and (2) the effect-size based meta-analysis using a fixed effect model (estimated by restricted maximum-likelihood) since there was no evidence of significant heterogeneity[11]. Similar outcome to "summation of p value" was obtained using the Fisher's test for combining p-values (data not shown). To conduct the meta-analysis we used the R packages metaphor and metap.

Pyrosequencing

A representative CpG within miR21 locus exhibiting significant DNA methylation differences in the Illumina 450K array (cg07181702) was selected for pyrosequencing validation. Primers were designed using PyroMark Design software (Qiagen) (Fwd: GAA ATG TTT GGG TTT TTT TGG TTT G, Rev: ACC CAT CCA CTA ATA TTA CCA TAA AAT TCA, sequencing: ATT CAA CAA TCA ACA TCA AT). Genomic DNA (200 ng) was bisulfite-converted using an EpiTect Bisulfite Kit (Qiagen), bisulfite-converted DNA was eluted in 20 µl of elution buffer and ~15 ng was applied as a template in the PCR performed with the PyroMarks PCR kit (Qiagen) using 5'-biotinylated forward primer. The entire PCR product, 4 pmol of the sequencing primer, and streptavidin sepharose high-performance beads (GR Healthcare), were used for pyrosequencing on the PSQ 96 system and PyroMark Gold 96 reagent kit (Qiagen). The PyroMark CpG software 1.0.11 (Qiagen) served for data analysis.

RNA extraction

For subsequent qPCR analysis total RNA from the discovery and independent sample cohort was isolated using standard TRIzol protocol (Invitrogen) and Allprep Total RNA/DNA Kit (Qiagen), respectively, according to manufacturer´s recommendations. RNA concentration and purity were determined by measurement of A260/A280 ratios with a NanoDrop ND-1000 Spectrophotometer (NanoDrop Technologies). For subsequent transcriptome analysis RNA, was extracted using the miRNeasy Kit (Qiagen) according to manufacturer´s recommendations. The RNA integrity was determined using Bioanalyzer (Agilent Technologies). All samples had an RNA integrity number (RIN) above 8. All RNA samples obtained from the different preparations were immediately frozen and stored at −80°C until further use.

Quantitative real-time PCR analysis

Expression levels of miR-21 were determined by quantitative real-time PCR (qRT-PCR) using TaqMan MicroRNA Assay Kit specific for mature miR-21 (No: 000397, Applied Biosystems) following the manufacturer's protocol. Expression levels were quantified by the CFX384 Real-Time PCR Detection System and analyzed using CFX manager software (Bio-Rad). Relative quantification of miRNA was quantified using the $2^{-\Delta\Delta CT}$ method and normalized against RNU48 for each sample. For *VMP1* expression detection cDNA was converted from RNA using the Bio-Rad iScript cDNA sythesis Kit (Bio-Rad). *VMP1* expression was accessed by qPCR (fwd 5'-GGT GCT GAA CCA AGA TGA-3'; rev 5'-GCA CTG TGT TGG CGT ACA G-3') using CFX384 Real-Time detection system and analyzed by CFX manager software (Bio-Rad). Cycling conditions were as follows 95C° for 2 min followed by 40 repeats at 95 C° for 20 s, 58C° for 20 s and 72C° for 30 s. *VMP1* expression was normalized to beta actin reference gene (fwd 5'-GAC TTC GAG CAA GAG ATG G-3' rev 5'-GCA CTG TGT TGG CGT ACA G-3') for every sample and quantified using the $2^{-\Delta\Delta CT}$ method. Statistical analysis between the groups was performed using Student's t-test (for two groups) and ANOVA with Bonferroni correction for selected groups (for more than two groups), and correlation between expression and methylation levels was assessed using Pearson's correlation test in GraphPad Prism 5 (GraphPad).

Genotyping

Allelic discrimination of rs8070345 (in the *VMP1* locus) was performed using a predesigned TaqMan SNP Genotyping Assay (Cat. No: 4351379, Applied Biosystems) according to the manufacturer's protocol. PCR amplification was done in a CFX384 Real-Time PCR Detection

System (Bio-Rad) and results were read on a QuantStudio ™ 7 Flex system (Applied Biosystems).

Association and correlation analysis

In the GOLDN cohort we used the *lmekin* function of the R *kinship* package to fit linear mixed effects models with the methylation β-value at each of the interrogated CpG sites as the outcome and the following predictors: SNP, age, sex, study site (Utah vs. Minnesota), current smoking (yes vs. no), body mass index, 4 principal components capturing T-cell purity[3] (as fixed effects), and family (as a random effect). We additionally fit linear regression models with the methylation β-value at each CpG site as the outcome and the non-genetic predictors, namely age, smoking, and BMI, and the T-cell purity principal components.

In the Multiple Sclerosis cohort we fit linear regression model with the methylation β-value at each of the interrogated CpG sites as the outcome and the following predictors: SNP, age and sex. We additionally fit linear regression model with the methylation β-value at each of the interrogated CpG sites as the outcome and the following predictors: age, sex, Multiple Sclerosis Severity Score (MSSS) and lymphocyte count. All analyses were done in Rcmd.

Transcriptome analysis

Total RNA (500ng) with RIN above 8.0, was subjected to the Illumina TruSeq Stranded mRNA Library Preparation Protocol with Dual Indexes (Cat. No: RS-122-2013, Illumina). Libraries were quantified using the Kapa Library Quantification Kit (Cat. No: KK4824, Illumina). Sequencing was carried out on the Illumina HiSeq 2500 to generate 75bp Paired End Data with an average of 10M reads above Q30. The sequence reads were mapped to hg19 reference with Tophat2[12] and HTSeq[13] was used to quantify counts-per-gene. The expression data from $CD4^+$

cells (34 samples) were normalized with Conditional Quantile Normalization (CQN) method[14] followed by batch reduction by ComBat[7]. Differential expression analysis was conducted using limma tool[8] in order to identify differences between disease types.

Target gene enrichment analysis

Genes identified as miR-21 targets in Jurkat T cells by RIP-Chip (Czepiel *et al.*, ISBN 978-90-367-6788-0) and genes predicted and experimentally validated to be miR-21 targets by TarBase7.0[15] were selected for further investigation. Deviation of up- and down-regulated miR-21 target genes from the expected ratio of up-and down-regulated genes in RNAseq analysis (for p-values of 0.1, 0.05 and 0.01) was calculated for both lists of miR-21 target genes using a Chi-squared test. Enrichment of up- and down-regulated targets among differentially expressed targets in RNAseq analysis (for p-values of 0.1, 0.05 and 0.01) was calculated for both lists of miR-21 target genes using the Fisher's exact test in GraphPad Prism 5 (GraphPad Software, San Diego, CA, USA). Similar results were obtained when predicted miR-21 target genes were based on being predicted by a minimum of the two of the following tools: miR-21 targets in Jurkat cells, miRTar, TarBase7.0, TargetScan7.1 and microT-CDS.

Ingenuity Pathways Analysis

Two list of genes, 1) differentially expressed genes between RR-MS and HC ($p<0.05$) and 2) TarBase7.0-predicted miR-21 target genes up-regulated in RR-MS compared to HC ($p<0.05$) from RNAseq analysis were uploaded to the Ingenuity Pathways Analysis platform (Qiagen). The first list was used to assess significant up-stream regulators that can explain observed gene expression changes estimated by the overlap p-value, which is calculated using Fisher's exact test and significance is generally attributed to $p<0.01$. The activation z-score is used to infer the

activation states of predicted upstream regulators and $z<-2$ and $z>2$ indicate significantly inhibited and activated upstream regulators, respectively. The second list of genes was used to infer biological functions that might be affected by miR-21. Right-tailed Fisher's exact test was used to calculate a p-value determining the probability that each biological function assigned to that data set is due to chance alone. Benjamini-Hochberg correction for multiple testing was used to calculate significant p-values.

References

1. Absher DM, Li X, Waite LL, et al. Genome-wide DNA methylation analysis of systemic lupus erythematosus reveals persistent hypomethylation of interferon genes and compositional changes to CD4+ T-cell populations. *PLoS genetics*. 2013; 9: e1003678.
2. Zhi D, Aslibekyan S, Irvin MR, et al. SNPs located at CpG sites modulate genome-epigenome interaction. *Epigenetics*. 2013; 8: 802-6.
3. Irvin MR, Zhi D, Joehanes R, et al. Epigenome-wide association study of fasting blood lipids in the Genetics of Lipid-lowering Drugs and Diet Network study. *Circulation*. 2014; 130: 565-72.
4. Aslibekyan S, Kabagambe EK, Irvin MR, et al. A genome-wide association study of inflammatory biomarker changes in response to fenofibrate treatment in the Genetics of Lipid Lowering Drug and Diet Network. *Pharmacogenetics and genomics*. 2012; 22: 191-7.
5. Marabita F, Almgren M, Lindholm ME, et al. An evaluation of analysis pipelines for DNA methylation profiling using the Illumina HumanMethylation450 BeadChip platform. *Epigenetics*. 2013; 8: 333-46.
6. Houseman EA, Accomando WP, Koestler DC, et al. DNA methylation arrays as surrogate measures of cell mixture distribution. *BMC bioinformatics*. 2012; 13: 86.
7. Johnson WE, Li C and Rabinovic A. Adjusting batch effects in microarray expression data using empirical Bayes methods. *Biostatistics*. 2007; 8: 118-27.
8. Ritchie ME, Phipson B, Wu D, et al. limma powers differential expression analyses for RNA-sequencing and microarray studies. *Nucleic acids research*. 2015; 43: e47.

9. Smyth GK. Linear models and empirical bayes methods for assessing differential expression in microarray experiments. *Statistical applications in genetics and molecular biology*. 2004; 3: Article3.

10. Edgington ES. An additive method for combining probability values from independent experiments. *J Psychol*. 1972; 80: 351-63.

11. Hedges LV and Olkin I. Statistical methods for meta-analysis. *San Diego: Academic Press*. 1985.

12. Kim D, Pertea G, Trapnell C, Pimentel H, Kelley R and Salzberg SL. TopHat2: accurate alignment of transcriptomes in the presence of insertions, deletions and gene fusions. *Genome biology*. 2013; 14: R36.

13. Anders S, Pyl PT and Huber W. HTSeq--a Python framework to work with high-throughput sequencing data. *Bioinformatics*. 2015; 31: 166-9.

14. Hansen KD, Irizarry RA and Wu Z. Removing technical variability in RNA-seq data using conditional quantile normalization. *Biostatistics*. 2012; 13: 204-16.

15. Vlachos IS, Paraskevopoulou MD, Karagkouni D, et al. DIANA-TarBase v7.0: indexing more than half a million experimentally supported miRNA:mRNA interactions. *Nucleic acids research*. 2015; 43: D153-9.
