# Supplementary material for: Hypermethylation of MIR21 in CD4+ T cells from patients with relapsing-remitting multiple sclerosis associates with lower miRNA-21 levels and concomitant up-regulation of its target genes
Source: Mult Scler. 2017 Aug 2;24(10):1288–300. doi: 10.1177/1352458517721356 (PMC5794671; doi:10.1177/1352458517721356)
Supplement: Supplementary material [file MSJ721356_supplementary_table_1.pdf]

Cohort	Status	Gender	Age	EDSS	MSSS	Lymphocytes	Treatment at sampling	Treatment prior to sampling	450K	RNAseq	miR-21qPCR
discovery	RR-MS remissio	F	37	3	2.91	1.7	never Tx		X	X	X
discovery	RR-MS remissio	F	40	0	0.67	1.8	never Tx		X	X	X
discovery	RR-MS remissio	M	44	1.5		1.8	never Tx		X	X	X
discovery	RR-MS remissio	M	40	0.5	2.44		never Tx			X	
discovery	RR-MS remissio	F	26	1	5.87	2.4	never Tx		X	X	X
discovery	RR-MS remissio	F	29	2	5.87	1.7	never Tx		X	X	X
discovery	RR-MS remissio	M	34	2	4.3		never Tx			X	
discovery	RR-MS remissio	F	35	1	1.13	2.2	no Tx	Fingolimod	X	X	X
discovery	RR-MS remissio	F	46	5	3.44	1.6	IVIg		X	X	X
discovery	RR-MS remissio	F	37	1.5	3.34	2.7	study drug		X		X
discovery	RR-MS remissio	M	29	1	0.88	1.6	no Tx	IFN	X	X	X
discovery	RR-MS remissio	M	32	3	3.05	2.7	no Tx	IFN	X	X	X
discovery	RR-MS remissio	F	57	2.5	4.13	1.9	no Tx	study drug	X		X
discovery	RR-MS remissio	F	41	2	0.71	1.6	never Tx		X		X
discovery	SP-MS	M	45	4	2.82	2.3	no Tx	IFN	X	X	
discovery	SP-MS	M	50	6.5	5.99	2.0	no Tx	study drug	X	X	X
discovery	SP-MS	M	56	6.5	5.99	2.2	never Tx		X	X	X
discovery	SP-MS	F	62	7	7.97		no Tx	IFN		X	
discovery	SP-MS	F	63	5		2.3	never Tx		X	X	X
discovery	SP-MS	F	56	8	8.75		no Tx	Copaxone, IFN		X	
discovery	SP-MS	F	35	5	7.32	1.8	no Tx	IFN, Copaxone	X	X	X
discovery	SP-MS	F	60	5	5.82	1.5	no Tx	IFN, Mitoxantrone	X	X	X
discovery	SP-MS	M	44	6	5.43	2.0	no Tx	IFN, study drug	X	X	X
discovery	SP-MS	F	53	6	6.01	1.3	no Tx	IFN, Mitoxantrone		X	
discovery	SP-MS	F	53	6.5	5.61	2.3	no Tx	IFN, study drug		X	
discovery	SP-MS	F	50	3.5	4.55	1.4	no Tx	IFN	X		X
discovery	HC	F	60						X	X	
discovery	HC	F	44						X		X
discovery	HC	M	31						X	X	
discovery	HC	F	35						X	X	
discovery	HC	M	31						X	X	X
discovery	HC	F	48						X	X	X
discovery	HC	F	28							X	
discovery	HC	M	30							X	
discovery	HC	F	30						X		X
discovery	HC	F	28						X		X
discovery	HC	F	27							X	
discovery	HC	F	32						X	X	X
discovery	HC	F	62						X	X	
discovery	HC	M	44						X	X	X
discovery	HC	M	54						X	X	X
**Summarized discovery cohort 450K; mean (range):**											
RR-MS		9F (75%) / 3M (25%)	38 (26-57)	2,0 (0-5,0)	2,9 (0,7-5,9)	2,0 (1,6-2,7)					
SP-MS		4F (50%) / 4F (50%)	50 (35-63)	5,2 (3,5-6,5)	5,4 (2,8-7,3)	1,9 (1,4-2,3)					
HC		8F (67%) / 4M (33%)	42 (28-62)	N/A	N/A	N/A					
**Summarized discovery cohort RNA-seq, mean (range):**											
RR-MS		6F (54%) / 5M (46%)	36 (26-46)	1,8 (0-5,0)	3,1 (0,7-5,9)	1,9 (1,6-2,7)					
SP-MS		7F (64%) / 4M (36%)	52 (35-63)	6,0 (4,0-8,0)	6,2 (2,8-8,8)	2,0 (1,3-2,3)					
HC		7F (58%) / 5M (42%)	40 (27-62)	N/A	N/A	N/A					
validation	CIS - RR-MS	F	41				never Tx				X
validation	CIS - RR-MS	F	50				never Tx				X
validation	RR-MS relapse	F	54				never Tx				X
validation	RR-MS relapse	F	38				never Tx				X
validation	RR-MS remissio	F	36				Avonex				X
validation	RR-MS remissio	F	33				never Tx				X
validation	RR-MS remissio	M	48				Rebif				X
validation	RR-MS remissio	F	34				never Tx				X
validation	RR-MS remissio	F	52				never Tx				X
validation	RR-MS remissio	F	31				never Tx				X
validation	RR-MS remissio	F	27				Avonex				X
validation	RR-MS remissio	F	73				never Tx				X
validation	RR-MS remissio	M	23				never Tx				X
validation	RR-MS remissio	M	37				never Tx				X
validation	RR-MS remissio	M	52				never Tx				X
validation	RR-MS remissio	M	36				Avonex				X
validation	RR-MS remissio	M	45				Avonex				X
validation	RR-MS remissio	F	29				never Tx				X
validation	RR-MS remissio	F	32				never Tx				X
validation	RR-MS remissio	F	29				never Tx				X
validation	RR-MS remissio	M	34				never Tx				X
validation	RR-MS remissio	F	40				never Tx				X
validation	RR-MS remissio	F	34				never Tx				X
validation	RR-MS remissio	F	27				Avonex				X
validation	RR-MS remissio	M	28				never Tx				X
validation	RR-MS remissio	F	31				never Tx				X
validation	RR-MS remissio	F	50				Avonex				X
validation	RR-MS remissio	F	35				Copaxone				X
validation	RR-MS remissio	F	31				no Tx	Avonex			X
validation	RR-MS remissio	F	24				Betaferon				X
validation	SP-MS	F	54				never Tx				X
validation	SP-MS	M	47				never Tx				X
validation	SP-MS	M	57				never Tx				X
validation	SP-MS	F	41				never Tx				X
validation	SP-MS	F	51				never Tx				X
validation	SP-MS	F	65				never Tx				X
validation	SP-MS	F	36				never Tx				X
validation	SP-MS	F	51				Study drug				X
validation	SP-MS	F	52				Avonex				X
validation	SP-MS	F	59				never Tx				X
validation	SP-MS	M	59				never Tx				X
validation	HC	F	22								X
validation	HC	F	23								X
validation	HC	M	35								X
validation	HC	M	28								X
validation	HC	F	33								X
validation	HC	M	35								X
validation	HC	M	23								X
validation	HC	M	21								X
validation	HC	F	22								X
validation	HC	F	34								X
validation	HC	M	30								X
validation	HC	M	40								X
validation	INDC	F	47				never Tx				X
validation	INDC	M	53				never Tx				X
validation	INDC	F	32				never Tx				X
validation	INDC	F	64				never Tx				X
validation	INDC	F	40				never Tx				X
validation	INDC	M	51				never Tx				X
validation	INDC	F	68				never Tx				X
validation	INDC	F	53				never Tx				X
validation	INDC	F	74				never Tx				X
Summarized validation Cohort											
RR-MS patients		22F (73%) / 8M (27%)	38 (23-73)								
SP-MS patients		8F (73%) / 3M (27%)	52 (36-65)								
HC		5F (42%) / 7M (58%)	29 (21-40)								
INDC		7F (78%) / 2M (22%)	54 (32-74)								
