# Supplementary material for: Hypermethylation of MIR21 in CD4+ T cells from patients with relapsing-remitting multiple sclerosis associates with lower miRNA-21 levels and concomitant up-regulation of its target genes
Source: Mult Scler. 2017 Aug 2;24(10):1288–300. doi: 10.1177/1352458517721356 (PMC5794671; doi:10.1177/1352458517721356)
Supplement: Supplementary material [file MSJ721356_supplementary_table_2.pdf]

Supplementary Table 2: Differential expression in CD4+ T cells of predicted miR-21 target genes between RR-MS and HC. Genes identified as miR-21 targets in Jurkat T cells by RIP-Chip and genes predicted and experimentally validated to be miR-21 targets by TarBase7.0 were selected for investigation.

Jurkat target ID	Gene Name	Description	logFC	AveExpr	P.Value
ENSG00000180667	**YOD1**	YOD1 deubiquitinase [Source:HGNC Symbol;Acc: HGNC:25035]	0.30	5.09	3.28E-04
ENSG00000100354	TNRC6B	trinucleotide repeat containing 6B [Source:HGNC Symbol;Acc: HGNC:29190]	0.13	6.71	4.99E-03
ENSG00000146676	**PURB**	purine rich element binding protein B [Source:HGNC Symbol;Acc: HGNC:9702]	0.17	5.75	9.29E-03
ENSG00000145715	RASA1	RAS p21 protein	0.17	6.59	1.23E-02

TarBase7.0 target ID	Gene Name	Description	logFC	AveExpr	P.Value
ENSG00000164284	GRPEL2	GrpE like 2, mitochondrial [Source:HGNC Symbol;Acc: HGNC:21060]	0.44	5.87	6.63E-05
ENSG00000180667	**YOD1**	YOD1 deubiquitinase [Source:HGNC Symbol;Acc: HGNC:25035]	0.30	5.09	3.28E-04
ENSG00000113369	ARRDC3	arrestin domain containing 3 [Source:HGNC Symbol;Acc: HGNC:29263]	0.44	8.17	7.68E-04
ENSG00000143753	DEGS1	delta 4-desaturase,	0.21	6.40	2.74E-03

		activator 1 [Source:HGNC Symbol;Acc: HGNC:9871]						sphingolipid 1 [Source:HGNC Symbol;Acc: HGNC:13709]			
ENSG00000110987	**BCL7A**	BCL tumor suppressor 7A [Source:HGNC Symbol;Acc: HGNC:1004]	0.63	2.13	2.20E-02	ENSG00000180979	LRRC57	leucine rich repeat containing 57 [Source:HGNC Symbol;Acc: HGNC:26719]	0.33	5.02	4.09E-03
ENSG00000197579	**TOPORS**	TOP1 binding arginine/serine rich protein [Source:HGNC Symbol;Acc: HGNC:21653]	0.12	5.20	3.33E-02	ENSG00000150753	CCT5	chaperonin containing TCP1 subunit 5 [Source:HGNC Symbol;Acc: HGNC:1618]	-0.17	6.06	4.97E-03
ENSG00000152518	ZFP36L2	ZFP36 ring finger protein like 2 [Source:HGNC Symbol;Acc: HGNC:1108]	0.18	11.05	3.64E-02	ENSG00000253719	ATXN7L3B	ataxin 7 like 3B [Source:HGNC Symbol;Acc: HGNC:37931]	0.17	8.07	5.66E-03

ENSG00000131023	**LATS1**	large tumor suppressor kinase 1 [Source:HGNC Symbol;Acc: HGNC:6514]	-0.07	5.87	3.88E-02
ENSG00000124209	**RAB22A**	RAB22A, member RAS oncogene family [Source:HGNC Symbol;Acc: HGNC:9764]	0.11	4.57	4.50E-02
ENSG00000138336	**TET1**	tet methylcytosine dioxygenase 1 [Source:HGNC Symbol;Acc: HGNC:29484]	-0.26	1.57	4.81E-02
ENSG00000076641	**PAG1**	phosphoprotein membrane anchor with glycosphingolipid microdomai	0.18	8.28	7.03E-02

ENSG00000131381	RBSN	rabenosyn, RAB effector [Source:HGNC Symbol;Acc: HGNC:20759]	0.13	5.61	7.10E-03
ENSG00000141582	CBX4	chromobox 4 [Source:HGNC Symbol;Acc: HGNC:1554]	0.22	6.52	9.03E-03
ENSG00000146676	**PURB**	purine rich element binding protein B [Source:HGNC Symbol;Acc: HGNC:9702]	0.17	5.75	9.29E-03
ENSG00000119844	AFTPH	aftiphilin [Source:HGNC Symbol;Acc: HGNC:25951]	-0.14	6.05	1.24E-02

		ns 1 [Source:HGNC Symbol;Acc:HGNC:30043]			
ENSG00000165244	**ZNF367**	zinc finger protein 367 [Source:HGNC Symbol;Acc:HGNC:18320]	0.16	3.85	7.29E-02
ENSG00000152520	PAN3	PAN3 poly(A) specific ribonuclease subunit [Source:HGNC Symbol;Acc:HGNC:29991]	0.09	7.10	7.60E-02
ENSG00000166225	**FRS2**	fibroblast growth factor receptor substrate 2 [Source:HGNC Symbol;Acc:HGNC:16971	0.08	4.97	9.50E-02

ENSG00000118402	ELOVL4	ELOVL fatty acid elongase 4 [Source:HGNC Symbol;Acc:HGNC:14415]	0.61	2.44	1.48E-02
ENSG00000164466	SFXN1	sideroflexin 1 [Source:HGNC Symbol;Acc:HGNC:16085]	0.14	6.23	1.49E-02
ENSG00000163629	PTPN13	protein tyrosine phosphatase, non-receptor type 13 [Source:HGNC Symbol;Acc:	0.67	3.10	1.55E-02

		]						HGNC:9646]			
ENSG00000171316	CHD7	chromodomain helicase DNA binding protein 7 [Source:HGNC Symbol;Acc: HGNC:20626]	0.14	5.57	1.10E-01	ENSG00000104643	MTMR9	myotubularin related protein 9 [Source:HGNC Symbol;Acc: HGNC:14596]	0.09	4.85	1.65E-02
ENSG00000107679	PLEKHA1	pleckstrin homology domain containing A1 [Source:HGNC Symbol;Acc: HGNC:14335]	-0.12	5.70	1.25E-01	ENSG00000145907	G3BP1	G3BP stress granule assembly factor 1 [Source:HGNC Symbol;Acc: HGNC:30292]	0.09	6.92	1.88E-02
ENSG00000171132	PRKCE	protein kinase C epsilon [Source:HGNC Symbol;Acc: HGNC:9401]	0.12	2.63	1.31E-01	ENSG00000196776	CD47	CD47 molecule [Source:HGNC Symbol;Acc: HGNC:1682]	0.09	8.47	1.95E-02
ENSG00000110848	CD69	CD69 molecule [Source:HGNC Symbol;Acc: HGNC:1694]	-0.19	10.25	1.42E-01	ENSG00000110987	**BCL7A**	BCL tumor suppressor 7A [Source:HGNC Symbol;Acc:	0.63	2.13	2.20E-02

ENSG00000155640	-	-	0.16	4.26	1.46E-01
ENSG00000174010	**KLHL15**	kelch like family member 15 [Source:HGNC Symbol;Acc:HGNC:29347]	0.12	4.55	1.48E-01
ENSG00000134698	**AGO4**	argonaute 4, RISC catalytic component [Source:HGNC Symbol;Acc:HGNC:18424]	0.17	5.75	1.65E-01
ENSG00000152601	MBNL1	muscleblind like splicing regulator 1 [Source:HGNC Symbol;Acc:	-0.06	9.23	1.86E-01

		HGNC:1004]			
ENSG00000070214	SLC44A1	solute carrier family 44 member 1 [Source:HGNC Symbol;Acc:HGNC:18798]	-0.13	3.78	2.27E-02
ENSG00000173698	ADGRG2	adhesion G protein-coupled receptor G2 [Source:HGNC Symbol;Acc:HGNC:4516]	-1.00	-1.29	2.30E-02
ENSG00000109787	KLF3	Kruppel like factor 3 [Source:HGNC Symbol;Acc:HGNC:16516]	0.17	7.88	2.33E-02
ENSG00000119778	ATAD2B	ATPase family, AAA domain containing 2B [Source:HG	-0.12	4.38	2.36E-02

		HGNC:6923]			
ENSG00000197323	**TRIM33**	tripartite motif containing 33 [Source:HGNC Symbol;Acc:HGNC:16290]	0.04	6.56	1.95E-01
ENSG00000138593	SECISBP2L	SECIS binding protein 2 like [Source:HGNC Symbol;Acc:HGNC:28997]	0.05	6.04	1.96E-01
ENSG00000162599	NFIA	nuclear factor I A [Source:HGNC Symbol;Acc:HGNC:7784]	0.13	1.27	2.43E-01
ENSG00000177189	**RPS6KA3**	ribosomal protein S6	0.06	7.43	2.83E-01

		NC Symbol;Acc:HGNC:29230]			
ENSG00000185658	BRWD1	bromodomain and WD repeat domain containing 1 [Source:HGNC Symbol;Acc:HGNC:12760]	0.10	6.29	2.41E-02
ENSG00000158079	PTPDC1	protein tyrosine phosphatase domain containing 1 [Source:HGNC Symbol;Acc:HGNC:30184]	-0.19	3.27	2.52E-02
ENSG00000096060	FKBP5	FK506 binding protein 5 [Source:HGNC Symbol;Acc:HGNC:3721]	0.31	6.88	2.56E-02
ENSG00000119899	SLC17A5	solute carrier	0.14	4.31	2.74E-02

		kinase A3 [Source:HGNC Symbol;Acc: HGNC:10432]			
ENSG00000188895	MSL1	male specific lethal 1 homolog [Source:HGNC Symbol;Acc: HGNC:27905]	0.07	7.40	2.87E-01
ENSG00000118922	KLF12	Kruppel like factor 12 [Source:HGNC Symbol;Acc: HGNC:6346]	0.05	7.01	2.98E-01
ENSG00000181690	**PLAG1**	PLAG1 zinc finger [Source:HGNC Symbol;Acc: HGNC:9045]	0.14	4.53	2.99E-01
ENSG00000162378	**ZYG11B**	zyg-11 family member B, cell cycle	0.05	5.21	3.05E-01

		family 17 member 5 [Source:HGNC Symbol;Acc: HGNC:10933]			
ENSG00000196459	TRAPPC2	trafficking protein particle complex 2 [Source:HGNC Symbol;Acc: HGNC:23068]	0.11	5.06	3.06E-02
ENSG00000100664	EIF5	eukaryotic translation initiation factor 5 [Source:HGNC Symbol;Acc: HGNC:3299]	0.13	7.55	3.10E-02
ENSG00000143013	LMO4	LIM domain only 4 [Source:HGNC Symbol;Acc: HGNC:6644]	0.33	4.30	3.20E-02
ENSG00000164190	NIPBL	NIPBL, cohesin loading	-0.07	6.49	3.25E-02

		regulator [Source:HGNC Symbol;Acc: HGNC:25820]			
ENSG00000204217	**BMPR2**	bone morphogenetic protein receptor type 2 [Source:HGNC Symbol;Acc: HGNC:1078]	0.06	4.33	3.20E-01
ENSG00000081014	AP4E1	adaptor related protein complex 4 epsilon 1 subunit [Source:HGNC Symbol;Acc: HGNC:573]	-0.04	4.62	3.29E-01
ENSG00000185009	**AP3M1**	adaptor related protein complex 3 mu 1 subunit [Source:HGNC Symbol;Acc:	-0.04	5.67	3.68E-01

		factor [Source:HGNC Symbol;Acc: HGNC:28862]			
ENSG00000048471	SNX29	sorting nexin 29 [Source:HGNC Symbol;Acc: HGNC:30542]	0.12	4.84	3.29E-02
ENSG00000197579	**TOPORS**	TOP1 binding arginine/serine rich protein [Source:HGNC Symbol;Acc: HGNC:21653]	0.12	5.20	3.33E-02
ENSG00000127337	YEATS4	YEATS domain containing 4 [Source:HGNC Symbol;Acc: HGNC:24859]	-0.25	5.66	3.42E-02

		HGNC:569]										
ENSG00000107864	**CPEB3**	cytoplasmic polyadenylation element binding protein 3 [Source:HGNC Symbol;Acc:HGNC:21746]	0.07	2.84	3.74E-01		ENSG00000196262	PPIA	peptidylprolyl isomerase A [Source:HGNC Symbol;Acc:HGNC:9253]	0.11	8.48	3.71E-02
ENSG00000150593	**PDCD4**	programmed cell death 4 (neoplastic transformation inhibitor) [Source:HGNC Symbol;Acc:HGNC:8763]	0.05	8.46	3.90E-01		ENSG00000092871	RFFL	ring finger and FYVE-like domain containing E3 ubiquitin protein ligase [Source:HGNC Symbol;Acc:HGNC:24821]	0.11	5.60	3.76E-02
ENSG00000143756	FBXO28	F-box protein 28 [Source:HGNC Symbol;Acc:HGNC:29046]	0.04	5.30	3.91E-01		ENSG00000131023	**LATS1**	large tumor suppressor kinase 1 [Source:HGNC Symbol;Acc:HGNC:6514]	-0.07	5.87	3.88E-02
ENSG00000182568	**SATB1**	SATB homeobox 1 [Source:HGN	0.09	7.64	4.06E-01		ENSG00000144909	OSBPL11	oxysterol binding protein like	-0.17	4.79	4.23E-02

		C Symbol;Acc: HGNC:10541]			
ENSG00000119866	**BCL11A**	B-cell CLL/lymphoma 11A [Source:HGNC Symbol;Acc: HGNC:13221]	-0.35	-0.92	4.47E-01
ENSG00000173281	**PPP1R3B**	protein phosphatase 1 regulatory subunit 3B [Source:HGNC Symbol;Acc: HGNC:14942]	0.09	3.82	4.58E-01
ENSG00000171940	**ZNF217**	zinc finger protein 217 [Source:HGNC Symbol;Acc: HGNC:13009]	0.03	6.68	5.29E-01
ENSG00000157933	**SKI**	SKI proto-oncogene [Source:HGN	0.05	7.14	5.44E-01

		11 [Source:HGNC Symbol;Acc: HGNC:16397]			
ENSG00000066933	MYO9A	myosin IXA [Source:HGNC Symbol;Acc: HGNC:7608]	-0.09	4.35	4.30E-02
ENSG00000118971	CCND2	cyclin D2 [Source:HGNC Symbol;Acc: HGNC:1583]	0.13	7.23	4.31E-02
ENSG00000169564	PCBP1	poly(rC) binding protein 1 [Source:HGNC Symbol;Acc: HGNC:8647]	-0.14	9.08	4.32E-02
ENSG00000167548	KMT2D	lysine methyltransferase 2D	-0.08	6.96	4.38E-02

		C Symbol;Acc: HGNC:10896]			
ENSG00000143878	**RHOB**	ras homolog family member B [Source:HGNC Symbol;Acc: HGNC:668]	-0.19	5.49	5.62E-01
ENSG00000102908	NFAT5	nuclear factor of activated T-cells 5 [Source:HGNC Symbol;Acc: HGNC:7774]	-0.03	5.84	5.65E-01
ENSG00000163939	**PBRM1**	polybromo 1 [Source:HGNC Symbol;Acc: HGNC:30064]	-0.02	5.89	5.65E-01
ENSG00000165572	**KBTBD6**	kelch repeat and BTB domain containing 6	-0.05	2.93	5.77E-01

		[Source:HGNC Symbol;Acc: HGNC:7133]			
ENSG00000125538	IL1B	interleukin 1 beta [Source:HGNC Symbol;Acc: HGNC:5992]	-1.38	0.88	4.39E-02
ENSG00000129128	SPCS3	signal peptidase complex subunit 3 [Source:HGNC Symbol;Acc: HGNC:26212]	0.10	7.47	4.41E-02
ENSG00000124209	**RAB22A**	RAB22A, member RAS oncogene family [Source:HGNC Symbol;Acc: HGNC:9764]	0.11	4.57	4.50E-02
ENSG00000138336	**TET1**	tet methylcytosine dioxygenase	-0.26	1.57	4.81E-02

		[Source:HGNC Symbol;Acc: HGNC:25340]			
ENSG00000155744	**FAM126B**	family with sequence similarity 126 member B [Source:HGNC Symbol;Acc: HGNC:28593]	0.03	4.63	6.01E-01
ENSG00000196937	**FAM3C**	family with sequence similarity 3 member C [Source:HGNC Symbol;Acc: HGNC:18664]	0.04	4.85	6.18E-01
ENSG00000123124	WWP1	WW domain containing E3 ubiquitin protein ligase 1 [Source:HGNC Symbol;Acc:	0.03	6.97	6.71E-01

		1 [Source:HGNC Symbol;Acc: HGNC:29484]			
ENSG00000153187	HNRNPU	heterogeneous nuclear ribonucleoprotein U [Source:HGNC Symbol;Acc: HGNC:5048]	-0.10	8.12	4.94E-02
ENSG00000185246	PRPF39	pre-mRNA processing factor 39 [Source:HGNC Symbol;Acc: HGNC:20314]	-0.08	6.85	5.31E-02
ENSG00000109458	GAB1	GRB2 associated binding protein 1 [Source:HGNC Symbol;Acc: HGNC:4066]	-0.83	-1.43	5.55E-02

		HGNC:17004]									
ENSG00000101972	**STAG2**	stromal antigen 2 [Source:HGNC Symbol;Acc: HGNC:11355]	0.01	6.93	6.78E-01	ENSG00000112159	MDN1	midasin AAA ATPase 1 [Source:HGNC Symbol;Acc: HGNC:18302]	-0.12	6.59	5.75E-02
ENSG00000204116	CHIC1	cysteine rich hydrophobic domain 1 [Source:HGNC Symbol;Acc: HGNC:1934]	0.02	5.46	6.97E-01	ENSG00000135046	ANXA1	annexin A1 [Source:HGNC Symbol;Acc: HGNC:533]	-0.29	8.85	5.78E-02
ENSG00000095015	**MAP3K1**	mitogen-activated protein kinase kinase kinase 1 [Source:HGNC Symbol;Acc: HGNC:6848]	0.03	8.20	7.24E-01	ENSG00000031691	CENPQ	centromere protein Q [Source:HGNC Symbol;Acc: HGNC:21347]	-0.25	3.52	6.30E-02
ENSG00000112739	PRPF4B	pre-mRNA processing factor 4B [Source:HGNC Symbol;Acc:	-0.01	7.52	7.66E-01	ENSG00000196227	FAM217B	family with sequence similarity 217 member B [Source:HG	0.12	5.04	6.53E-02

		HGNC:17346]						NC Symbol;Acc: HGNC:1617 0]			
ENSG0000015774 1	UBN2	ubinuclein 2 [Source:HGN C Symbol;Acc: HGNC:21931]	- 0.02	5.00	7.75E-01	ENSG00000154518	ATP5G3	ATP synthase, H+ transporting , mitochondri al Fo complex subunit C3 (subunit 9) [Source:HG NC Symbol;Acc: HGNC:843]	0.10	4.69	6.92E-02
ENSG0000011330 0	**CNOT6**	CCR4-NOT transcription complex subunit 6 [Source:HGN C Symbol;Acc: HGNC:14099]	0.02	5.64	7.85E-01	ENSG00000048649	RSF1	remodeling and spacing factor 1 [Source:HG NC Symbol;Acc: HGNC:1811 8]	0.07	5.37	6.93E-02
ENSG0000012892 3	**FAM63B**	family with sequence similarity 63 member B [Source:HGN C	0.02	4.99	8.39E-01	ENSG00000076641	**PAG1**	phosphopro tein membrane anchor with glycosphing olipid	0.18	8.28	7.03E-02

		Symbol;Acc: HGNC:26954]						microdomains 1 [Source:HGNC Symbol;Acc: HGNC:30043]			
ENSG00000105810	CDK6	cyclin dependent kinase 6 [Source:HGNC Symbol;Acc: HGNC:1777]	0.02	5.82	8.84E-01	ENSG00000169967	MAP3K2	mitogen-activated protein kinase kinase kinase 2 [Source:HGNC Symbol;Acc: HGNC:6854]	0.06	6.44	7.07E-02
ENSG00000138081	**FBXO11**	F-box protein 11 [Source:HGNC Symbol;Acc: HGNC:13590]	0.00	6.64	8.93E-01	ENSG00000165244	**ZNF367**	zinc finger protein 367 [Source:HGNC Symbol;Acc: HGNC:18320]	0.16	3.85	7.29E-02
ENSG00000178177	LCORL	ligand dependent nuclear receptor corepressor like [Source:HGNC Symbol;Acc:	0.01	2.93	9.10E-01	ENSG00000140299	BNIP2	BCL2 interacting protein 2 [Source:HGNC Symbol;Acc: HGNC:1083]	0.07	6.14	7.31E-02

		HGNC:30776]									
ENSG00000148730	**EIF4EBP2**	eukaryotic translation initiation factor 4E binding protein 2 [Source:HGNC Symbol;Acc: HGNC:3289]	0.00	7.12	9.40E-01	ENSG00000143614	GATAD2B	GATA zinc finger domain containing 2B [Source:HGNC Symbol;Acc: HGNC:30778]	0.06	6.47	7.94E-02
ENSG00000103064	SLC7A6	solute carrier family 7 member 6 [Source:HGNC Symbol;Acc: HGNC:11064]	0.01	8.52	9.45E-01	ENSG00000163624	CDS1	CDP-diacylglycerol synthase 1 [Source:HGNC Symbol;Acc: HGNC:1800]	-0.27	0.83	8.08E-02
ENSG00000115020	PIKFYVE	phosphoinositide kinase, FYVE-type zinc finger containing [Source:HGNC Symbol;Acc: HGNC:23785]	0.00	6.42	9.73E-01	ENSG00000183508	FAM46C	family with sequence similarity 46 member C [Source:HGNC Symbol;Acc: HGNC:24712]	-0.44	6.75	8.49E-02
ENSG00000145675	PIK3R1	phosphoinositide-3-	0.00	7.40	9.80E-01	ENSG00000008277	ADAM22	ADAM metallopept	-0.23	1.73	8.58E-02

		kinase regulatory subunit 1 [Source:HGNC Symbol;Acc:HGNC:8979]			
ENSG00000123472	ATPAF1	ATP synthase mitochondrial F1 complex assembly factor 1 [Source:HGNC Symbol;Acc:HGNC:18803]	0.00	5.17	9.89E-01
ENSG00000172575	**RASGRP1**	RAS guanyl releasing protein 1 [Source:HGNC Symbol;Acc:HGNC:9878]	0.00	8.41	9.99E-01

		idase domain 22 [Source:HGNC Symbol;Acc:HGNC:201]			
ENSG00000196591	HDAC2	histone deacetylase 2 [Source:HGNC Symbol;Acc:HGNC:4853]	-0.13	4.32	8.59E-02
ENSG00000080546	SESN1	sestrin 1 [Source:HGNC Symbol;Acc:HGNC:21595]	0.25	6.65	8.70E-02
ENSG00000198855	FICD	FIC domain containing [Source:HGNC Symbol;Acc:HGNC:18416]	0.21	2.08	9.01E-02

ENSG00000140575	IQGAP1	IQ motif containing GTPase activating protein 1 [Source:HGNC Symbol;Acc:HGNC:6110]	-0.13	6.82	9.03E-02
ENSG00000104343	UBE2W	ubiquitin conjugating enzyme E2 W (putative) [Source:HGNC Symbol;Acc:HGNC:25616]	0.08	4.35	9.23E-02
ENSG00000166225	**FRS2**	fibroblast growth factor receptor substrate 2 [Source:HGNC Symbol;Acc:HGNC:16971]	0.08	4.97	9.50E-02
ENSG00000118263	KLF7	Kruppel like factor 7 [Source:HGNC	0.16	6.27	9.58E-02

		Symbol;Acc:HGNC:6350]			
ENSG00000102218	RP2	retinitis pigmentosa 2 (X-linked recessive) [Source:HGNC Symbol;Acc:HGNC:10274]	-0.15	3.81	9.83E-02
ENSG00000168807	SNTB2	syntrophin beta 2 [Source:HGNC Symbol;Acc:HGNC:11169]	0.19	4.62	1.03E-01
ENSG00000025796	SEC63	SEC63 homolog, protein translocation regulator [Source:HGNC Symbol;Acc:HGNC:21082]	0.06	5.69	1.03E-01
ENSG00000107036	RIC1	RIC1 homolog, RAB6A GEF complex partner 1	0.07	5.43	1.04E-01

		[Source:HGNC Symbol;Acc:HGNC:17686]			
ENSG00000120696	KBTBD7	kelch repeat and BTB domain containing 7 [Source:HGNC Symbol;Acc:HGNC:25266]	0.29	2.02	1.08E-01
ENSG00000164916	FOXK1	forkhead box K1 [Source:HGNC Symbol;Acc:HGNC:23480]	0.12	5.91	1.13E-01
ENSG00000136045	PWP1	PWP1 homolog, endonuclein [Source:HGNC Symbol;Acc:HGNC:17015]	-0.07	6.17	1.14E-01
ENSG00000164039	BDH2	3-hydroxybutyrate dehydrogen	0.17	4.17	1.16E-01

		ase, type 2 [Source:HGNC Symbol;Acc:HGNC:32389]			
ENSG00000186687	LYRM7	LYR motif containing 7 [Source:HGNC Symbol;Acc:HGNC:28072]	-0.10	5.42	1.17E-01
ENSG00000065613	SLK	STE20 like kinase [Source:HGNC Symbol;Acc:HGNC:11088]	-0.08	6.04	1.24E-01
ENSG00000019995	ZRANB1	zinc finger RANBP2-type containing 1 [Source:HGNC Symbol;Acc:HGNC:18224]	0.06	5.57	1.25E-01
ENSG00000156735	BAG4	BCL2 associated athanogene 4	0.11	4.01	1.27E-01

		[Source:HGNC Symbol;Acc:HGNC:940]			
ENSG00000157106	SMG1	SMG1, nonsense mediated mRNA decay associated PI3K related kinase [Source:HGNC Symbol;Acc:HGNC:30045]	-0.08	7.37	1.36E-01
ENSG00000197147	LRRC8B	leucine rich repeat containing 8 family member B [Source:HGNC Symbol;Acc:HGNC:30692]	0.14	4.83	1.36E-01
ENSG00000090104	RGS1	regulator of G-protein signaling 1 [Source:HGNC Symbol;Acc:	-0.45	8.05	1.37E-01

		HGNC:9991]			
ENSG00000165219	GAPVD1	GTPase activating protein and VPS9 domains 1 [Source:HGNC Symbol;Acc:HGNC:23375]	0.07	5.09	1.37E-01
ENSG00000164237	CMBL	carboxymethylenebutenolidase homolog [Source:HGNC Symbol;Acc:HGNC:25090]	-0.37	0.54	1.40E-01
ENSG00000198743	SLC5A3	solute carrier family 5 member 3 [Source:HGNC Symbol;Acc:HGNC:11038]	-0.16	5.06	1.40E-01
ENSG00000174718	KIAA1551	KIAA1551 [Source:HGNC Symbol;Acc:	-0.13	8.85	1.40E-01

		HGNC:25559]			
ENSG00000120868	APAF1	apoptotic peptidase activating factor 1 [Source:HGNC Symbol;Acc:HGNC:576]	-0.08	5.15	1.43E-01
ENSG00000101856	PGRMC1	progesterone receptor membrane component 1 [Source:HGNC Symbol;Acc:HGNC:16090]	0.08	5.17	1.45E-01
ENSG00000155640	-	-	0.16	4.26	1.46E-01
ENSG00000151553	FAM160B1	family with sequence similarity 160 member B1 [Source:HGNC Symbol;Acc:HGNC:29320]	0.18	5.76	1.46E-01
ENSG00000174010	**KLHL15**	kelch like	0.12	4.55	1.48E-

		family member 15 [Source:HGNC Symbol;Acc:HGNC:29347]			01
ENSG00000153250	RBMS1	RNA binding motif single stranded interacting protein 1 [Source:HGNC Symbol;Acc:HGNC:9907]	0.10	6.79	1.48E-01
ENSG00000145604	SKP2	S-phase kinase-associated protein 2, E3 ubiquitin protein ligase [Source:HGNC Symbol;Acc:HGNC:10901]	-0.12	4.79	1.55E-01
ENSG00000111300	NAA25	N(alpha)-acetyltransferase 25, NatB auxiliary	0.08	5.08	1.58E-01

		subunit [Source:HGNC Symbol;Acc:HGNC:25783]			
ENSG00000100422	CERK	ceramide kinase [Source:HGNC Symbol;Acc:HGNC:19256]	0.09	7.87	1.59E-01
ENSG00000134698	**AGO4**	argonaute 4, RISC catalytic component [Source:HGNC Symbol;Acc:HGNC:18424]	0.17	5.75	1.65E-01
ENSG00000164252	AGGF1	angiogenic factor with G-patch and FHA domains 1 [Source:HGNC Symbol;Acc:HGNC:24684]	-0.06	5.64	1.65E-01
ENSG00000079785	DDX1	DEAD/H-	-	6.10	1.66E-

		box helicase 1 [Source:HGNC Symbol;Acc:HGNC:2734]	0.06		01
ENSG00000135318	NT5E	5'-nucleotidase ecto [Source:HGNC Symbol;Acc:HGNC:8021]	-0.43	2.73	1.71E-01
ENSG00000116106	EPHA4	EPH receptor A4 [Source:HGNC Symbol;Acc:HGNC:3388]	0.24	5.25	1.74E-01
ENSG00000125430	HS3ST3B1	heparan sulfate-glucosamine 3-sulfotransferase 3B1 [Source:HGNC Symbol;Acc:HGNC:5198]	0.11	5.43	1.76E-01
ENSG00000119138	KLF9	Kruppel like factor 9 [Source:HGNC	0.18	7.60	1.81E-01

		Symbol;Acc:HGNC:1123]			
ENSG00000165929	TC2N	tandem C2 domains, nuclear [Source:HGNC Symbol;Acc:HGNC:19859]	-0.11	8.46	1.82E-01
ENSG00000110422	HIPK3	homeodomain interacting protein kinase 3 [Source:HGNC Symbol;Acc:HGNC:4915]	-0.08	6.58	1.84E-01
ENSG00000118762	PKD2	polycystin 2, transient receptor potential cation channel [Source:HGNC Symbol;Acc:HGNC:9009]	0.18	4.94	1.86E-01
ENSG00000182504	CEP97	centrosomal protein 97 [Source:HGNC	0.07	4.48	1.90E-01

		Symbol;Acc: HGNC:26244]			
ENSG00000084093	REST	RE1 silencing transcription factor [Source:HGNC Symbol;Acc: HGNC:9966]	0.05	6.04	1.92E-01
ENSG00000156531	PHF6	PHD finger protein 6 [Source:HGNC Symbol;Acc: HGNC:18145]	-0.07	4.94	1.93E-01
ENSG00000132912	DCTN4	dynactin subunit 4 [Source:HGNC Symbol;Acc: HGNC:15518]	0.06	5.33	1.95E-01
ENSG00000197323	**TRIM33**	tripartite motif containing 33 [Source:HGNC Symbol;Acc: HGNC:1629	0.04	6.56	1.95E-01

		0]			
ENSG00000052795	FNIP2	folliculin interacting protein 2 [Source:HGNC Symbol;Acc:HGNC:29280]	-0.07	3.32	1.97E-01
ENSG00000211455	STK38L	serine/threonine kinase 38 like [Source:HGNC Symbol;Acc:HGNC:17848]	-0.06	4.69	1.99E-01
ENSG00000101665	SMAD7	SMAD family member 7 [Source:HGNC Symbol;Acc:HGNC:6773]	-0.28	4.63	2.08E-01
ENSG00000103769	RAB11A	RAB11A, member RAS oncogene family [Source:HGNC Symbol;Acc:	-0.05	5.76	2.12E-01

		HGNC:9760]			
ENSG00000141034	GID4	GID complex subunit 4 homolog [Source:HGNC Symbol;Acc:HGNC:28453]	-0.14	4.12	2.13E-01
ENSG00000172915	NBEA	neurobeachin [Source:HGNC Symbol;Acc:HGNC:7648]	0.23	2.11	2.16E-01
ENSG00000106780	MEGF9	multiple EGF like domains 9 [Source:HGNC Symbol;Acc:HGNC:3234]	0.11	5.54	2.18E-01
ENSG00000156052	GNAQ	G protein subunit alpha q [Source:HGNC Symbol;Acc:HGNC:4390]	0.05	6.60	2.18E-01
ENSG00000095139	ARCN1	archain 1 [Source:HGNC	-0.08	7.02	2.20E-01

		Symbol;Acc:HGNC:649]			
ENSG00000145916	RMND5B	required for meiotic nuclear division 5 homolog B [Source:HGNC Symbol;Acc:HGNC:26181]	-0.06	4.33	2.22E-01
ENSG00000106692	FKTN	fukutin [Source:HGNC Symbol;Acc:HGNC:3622]	0.08	5.14	2.26E-01
ENSG00000188647	PTAR1	protein prenyltransferase alpha subunit repeat containing 1 [Source:HGNC Symbol;Acc:HGNC:30449]	0.05	6.78	2.32E-01
ENSG00000101126	ADNP	activity dependent neuroprotector homeobox	0.05	6.66	2.35E-01

		[Source:HGNC Symbol;Acc:HGNC:15766]			
ENSG00000165102	HGSNAT	heparan-alpha-glucosaminide N-acetyltransferase [Source:HGNC Symbol;Acc:HGNC:26527]	0.07	5.52	2.38E-01
ENSG00000197111	PCBP2	poly(rC) binding protein 2 [Source:HGNC Symbol;Acc:HGNC:8648]	0.05	6.83	2.43E-01
ENSG00000136986	DERL1	derlin 1 [Source:HGNC Symbol;Acc:HGNC:28454]	-0.07	5.60	2.47E-01
ENSG00000101367	MAPRE1	microtubule associated protein RP/EB	-0.05	7.41	2.50E-01

		family member 1 [Source:HGNC Symbol;Acc:HGNC:6890]			
ENSG00000183808	RBM12B	RNA binding motif protein 12B [Source:HGNC Symbol;Acc:HGNC:32310]	-0.08	4.97	2.53E-01
ENSG00000138430	OLA1	Obg-like ATPase 1 [Source:HGNC Symbol;Acc:HGNC:28833]	0.06	5.00	2.54E-01
ENSG00000136381	IREB2	iron responsive element binding protein 2 [Source:HGNC Symbol;Acc:HGNC:6115]	0.05	5.96	2.57E-01
ENSG00000115183	TANC1	tetratricopeptide repeat,	0.29	-0.39	2.57E-01

		ankyrin repeat and coiled-coil containing 1 [Source:HGNC Symbol;Acc:HGNC:29364]			
ENSG00000013503	POLR3B	RNA polymerase III subunit B [Source:HGNC Symbol;Acc:HGNC:30348]	-0.10	3.60	2.61E-01
ENSG00000100030	MAPK1	mitogen-activated protein kinase 1 [Source:HGNC Symbol;Acc:HGNC:6871]	-0.06	6.48	2.62E-01
ENSG00000066117	SMARCD1	SWI/SNF related, matrix associated, actin dependent regulator of chromatin,	0.04	6.43	2.66E-01

		subfamily d, member 1 [Source:HGNC Symbol;Acc:HGNC:11106]			
ENSG00000155850	SLC26A2	solute carrier family 26 member 2 [Source:HGNC Symbol;Acc:HGNC:10994]	0.05	4.32	2.67E-01
ENSG00000129636	ITFG1	integrin alpha FG-GAP repeat containing 1 [Source:HGNC Symbol;Acc:HGNC:30697]	0.06	3.57	2.67E-01
ENSG00000165943	MOAP1	modulator of apoptosis 1 [Source:HGNC Symbol;Acc:HGNC:16658]	0.09	7.87	2.68E-01

ENSG00000135452	TSPAN31	tetraspanin 31 [Source:HGNC Symbol;Acc:HGNC:10539]	-0.10	4.22	2.70E-01
ENSG00000256223	ZNF10	zinc finger protein 10 [Source:HGNC Symbol;Acc:HGNC:12879]	-0.07	4.06	2.71E-01
ENSG00000120708	TGFBI	transforming growth factor beta induced [Source:HGNC Symbol;Acc:HGNC:11771]	-0.33	1.53	2.73E-01
ENSG00000177189	**RPS6KA3**	ribosomal protein S6 kinase A3 [Source:HGNC Symbol;Acc:HGNC:10432]	0.06	7.43	2.83E-01
ENSG00000156875	MFSD14A	major facilitator	0.05	6.70	2.84E-01

		superfamily domain containing 14A [Source:HGNC Symbol;Acc:HGNC:23363]			
ENSG00000112394	SLC16A10	solute carrier family 16 member 10 [Source:HGNC Symbol;Acc:HGNC:17027]	0.16	4.08	2.86E-01
ENSG00000144959	NCEH1	neutral cholesterol ester hydrolase 1 [Source:HGNC Symbol;Acc:HGNC:29260]	-0.14	2.41	2.91E-01
ENSG00000177200	CHD9	chromodomain helicase DNA binding protein 9 [Source:HG	0.08	4.59	2.92E-01

		NC Symbol;Acc: HGNC:25701]			
ENSG00000177932	ZNF354C	zinc finger protein 354C [Source:HGNC Symbol;Acc: HGNC:16736]	0.06	4.99	2.95E-01
ENSG00000134318	ROCK2	Rho associated coiled-coil containing protein kinase 2 [Source:HGNC Symbol;Acc: HGNC:10252]	-0.05	4.86	2.95E-01
ENSG00000180530	NRIP1	nuclear receptor interacting protein 1 [Source:HGNC Symbol;Acc: HGNC:8001]	0.08	5.14	2.97E-01
ENSG00000100916	BRMS1L	breast cancer	-0.07	3.53	2.98E-01

		metastasis-suppressor 1-like [Source:HGNC Symbol;Acc:HGNC:20512]			
ENSG00000130396	AFDN	afadin, adherens junction formation factor [Source:HGNC Symbol;Acc:HGNC:7137]	0.24	3.44	2.99E-01
ENSG00000181690	**PLAG1**	PLAG1 zinc finger [Source:HGNC Symbol;Acc:HGNC:9045]	0.14	4.53	2.99E-01
ENSG00000170860	LSM3	LSM3 homolog, U6 small nuclear RNA and mRNA degradation associated [Source:HGNC Symbol;Acc:	0.07	5.10	3.00E-01

		HGNC:17874]			
ENSG00000197329	PELI1	pellino E3 ubiquitin protein ligase 1 [Source:HGNC Symbol;Acc:HGNC:8827]	0.11	7.00	3.02E-01
ENSG00000101752	MIB1	mindbomb E3 ubiquitin protein ligase 1 [Source:HGNC Symbol;Acc:HGNC:21086]	0.07	5.54	3.04E-01
ENSG00000162378	**ZYG11B**	zyg-11 family member B, cell cycle regulator [Source:HGNC Symbol;Acc:HGNC:25820]	0.05	5.21	3.05E-01
ENSG00000099942	CRKL	CRK like proto-oncogene, adaptor	-0.07	6.49	3.07E-01

		protein [Source:HGNC Symbol;Acc:HGNC:2363]			
ENSG00000087448	KLHL42	kelch like family member 42 [Source:HGNC Symbol;Acc:HGNC:29252]	-0.08	5.27	3.10E-01
ENSG00000153944	MSI2	musashi RNA binding protein 2 [Source:HGNC Symbol;Acc:HGNC:18585]	0.05	5.89	3.17E-01
ENSG00000110047	EHD1	EH domain containing 1 [Source:HGNC Symbol;Acc:HGNC:3242]	-0.07	6.61	3.19E-01
ENSG00000204217	**BMPR2**	bone morphogenetic protein receptor type 2 [Source:HG	0.06	4.33	3.20E-01

		NC Symbol;Acc: HGNC:1078]			
ENSG00000154114	TBCEL	tubulin folding cofactor E like [Source:HGNC Symbol;Acc: HGNC:28115]	0.07	4.51	3.23E-01
ENSG00000086200	IPO11	importin 11 [Source:HGNC Symbol;Acc: HGNC:20628]	0.05	3.32	3.26E-01
ENSG00000074603	DPP8	dipeptidyl peptidase 8 [Source:HGNC Symbol;Acc: HGNC:16490]	0.03	5.74	3.29E-01
ENSG00000106415	GLCCI1	glucocorticoid induced 1 [Source:HGNC Symbol;Acc: HGNC:18713]	0.08	5.73	3.32E-01
ENSG00000160199	PKNOX1	PBX/knotte	-	3.79	3.37E-

		d 1 homeobox 1 [Source:HGNC Symbol;Acc:HGNC:9022]	0.05		01
ENSG00000072501	SMC1A	structural maintenance of chromosomes 1A [Source:HGNC Symbol;Acc:HGNC:11111]	-0.10	6.25	3.37E-01
ENSG00000060339	CCAR1	cell division cycle and apoptosis regulator 1 [Source:HGNC Symbol;Acc:HGNC:24236]	0.04	7.30	3.45E-01
ENSG00000157500	APPL1	adaptor protein, phosphotyrosine interacting with PH domain and	-0.04	5.21	3.54E-01

		leucine zipper 1 [Source:HGNC Symbol;Acc:HGNC:24035]			
ENSG00000171365	CLCN5	chloride voltage-gated channel 5 [Source:HGNC Symbol;Acc:HGNC:2023]	-0.10	1.88	3.60E-01
ENSG00000143575	HAX1	HCLS1 associated protein X-1 [Source:HGNC Symbol;Acc:HGNC:16915]	-0.06	6.87	3.63E-01
ENSG00000116954	RRAGC	Ras related GTP binding C [Source:HGNC Symbol;Acc:HGNC:19902]	-0.07	4.65	3.65E-01
ENSG00000185009	**AP3M1**	adaptor related	-0.04	5.67	3.68E-01

		protein complex 3 mu 1 subunit [Source:HGNC Symbol;Acc:HGNC:569]			
ENSG00000041353	RAB27B	RAB27B, member RAS oncogene family [Source:HGNC Symbol;Acc:HGNC:9767]	0.17	2.88	3.69E-01
ENSG00000107864	**CPEB3**	cytoplasmic polyadenylation element binding protein 3 [Source:HGNC Symbol;Acc:HGNC:21746]	0.07	2.84	3.74E-01
ENSG00000075239	ACAT1	acetyl-CoA acetyltransferase 1 [Source:HGNC	-0.08	3.93	3.75E-01

		Symbol;Acc:HGNC:93]			
ENSG00000068305	MEF2A	myocyte enhancer factor 2A [Source:HGNC Symbol;Acc:HGNC:6993]	0.06	5.71	3.79E-01
ENSG00000186432	KPNA4	karyopherin subunit alpha 4 [Source:HGNC Symbol;Acc:HGNC:6397]	-0.04	5.45	3.82E-01
ENSG00000163069	SGCB	sarcoglycan beta [Source:HGNC Symbol;Acc:HGNC:10806]	-0.08	3.39	3.83E-01
ENSG00000154305	MIA3	MIA family member 3, ER export factor [Source:HGNC Symbol;Acc:HGNC:24008]	-0.04	5.87	3.85E-01
ENSG00000087470	DNM1L	dynamin 1	0.03	5.96	3.90E-

		like [Source:HGNC Symbol;Acc:HGNC:2973]			01
ENSG00000150593	**PDCD4**	programmed cell death 4 (neoplastic transformation inhibitor) [Source:HGNC Symbol;Acc:HGNC:8763]	0.05	8.46	3.90E-01
ENSG00000152642	GPD1L	glycerol-3-phosphate dehydrogenase 1-like [Source:HGNC Symbol;Acc:HGNC:28956]	-0.07	5.84	3.95E-01
ENSG00000170500	LONRF2	LON peptidase N-terminal domain and ring finger 2 [Source:HGNC Symbol;Acc:	-0.28	-0.67	3.97E-01

		HGNC:24788]			
ENSG00000141367	CLTC	clathrin heavy chain [Source:HGNC Symbol;Acc:HGNC:2092]	-0.03	5.84	3.98E-01
ENSG00000152944	MED21	mediator complex subunit 21 [Source:HGNC Symbol;Acc:HGNC:11473]	0.06	5.65	3.99E-01
ENSG00000020577	SAMD4A	sterile alpha motif domain containing 4A [Source:HGNC Symbol;Acc:HGNC:23023]	-0.20	-0.19	4.00E-01
ENSG00000138600	SPPL2A	signal peptide peptidase like 2A [Source:HGNC Symbol;Acc:	0.06	5.57	4.02E-01

		HGNC:30227]			
ENSG00000182568	**SATB1**	SATB homeobox 1 [Source:HGNC Symbol;Acc:HGNC:10541]	0.09	7.64	4.06E-01
ENSG00000141759	TXNL4A	thioredoxin like 4A [Source:HGNC Symbol;Acc:HGNC:30551]	0.05	5.67	4.08E-01
ENSG00000058063	ATP11B	ATPase phospholipid transporting 11B (putative) [Source:HGNC Symbol;Acc:HGNC:13553]	-0.03	7.06	4.10E-01
ENSG00000127870	RNF6	ring finger protein 6 [Source:HGNC Symbol;Acc:	0.04	5.79	4.12E-01

		HGNC:10069]			
ENSG00000140525	FANCI	Fanconi anemia complementation group I [Source:HGNC Symbol;Acc:HGNC:25568]	-0.07	3.36	4.16E-01
ENSG00000178691	SUZ12	SUZ12 polycomb repressive complex 2 subunit [Source:HGNC Symbol;Acc:HGNC:17101]	-0.04	6.13	4.19E-01
ENSG00000007168	PAFAH1B1	platelet activating factor acetylhydrolase 1b regulatory subunit 1 [Source:HGNC Symbol;Acc:HGNC:8574]	0.03	7.22	4.20E-01

ENSG00000165650	PDZD8	PDZ domain containing 8 [Source:HGNC Symbol;Acc:HGNC:26974]	-0.08	5.11	4.24E-01
ENSG00000197969	VPS13A	vacuolar protein sorting 13 homolog A [Source:HGNC Symbol;Acc:HGNC:1908]	-0.05	6.20	4.26E-01
ENSG00000142599	RERE	arginine-glutamic acid dipeptide repeats [Source:HGNC Symbol;Acc:HGNC:9965]	0.05	6.56	4.32E-01
ENSG00000146281	PM20D2	peptidase M20 domain containing 2 [Source:HGNC Symbol;Acc:HGNC:21408]	-0.04	5.20	4.33E-01

ENSG00000058056	USP13	ubiquitin specific peptidase 13 (isopeptidase T-3) [Source:HGNC Symbol;Acc:HGNC:12611]	-0.06	3.82	4.35E-01
ENSG00000163513	TGFBR2	transforming growth factor beta receptor 2 [Source:HGNC Symbol;Acc:HGNC:11773]	0.06	8.68	4.35E-01
ENSG00000132463	GRSF1	G-rich RNA sequence binding factor 1 [Source:HGNC Symbol;Acc:HGNC:4610]	0.04	5.80	4.36E-01
ENSG00000118816	CCNI	cyclin I [Source:HGNC Symbol;Acc:HGNC:1595]	0.04	9.14	4.37E-01

ENSG00000158604	TMED4	transmembrane p24 trafficking protein 4 [Source:HGNC Symbol;Acc:HGNC:22301]	0.03	7.80	4.37E-01
ENSG00000180011	ZADH2	zinc binding alcohol dehydrogenase domain containing 2 [Source:HGNC Symbol;Acc:HGNC:28697]	0.06	5.30	4.37E-01
ENSG00000119866	**BCL11A**	B-cell CLL/lymphoma 11A [Source:HGNC Symbol;Acc:HGNC:13221]	-0.35	-0.92	4.47E-01
ENSG00000196914	ARHGEF12	Rho guanine nucleotide exchange factor 12 [Source:HGNC	0.17	2.66	4.49E-01

		Symbol;Acc: HGNC:14193]			
ENSG00000148158	SNX30	sorting nexin family member 30 [Source:HGNC Symbol;Acc: HGNC:23685]	-0.06	4.81	4.50E-01
ENSG00000060749	QSER1	glutamine and serine rich 1 [Source:HGNC Symbol;Acc: HGNC:26154]	0.05	4.39	4.53E-01
ENSG00000173281	**PPP1R3B**	protein phosphatase 1 regulatory subunit 3B [Source:HGNC Symbol;Acc: HGNC:14942]	0.09	3.82	4.58E-01
ENSG00000108021	FAM208B	family with sequence similarity 208	-0.03	6.22	4.69E-01

		member B [Source:HGNC Symbol;Acc:HGNC:23484]			
ENSG00000132842	AP3B1	adaptor related protein complex 3 beta 1 subunit [Source:HGNC Symbol;Acc:HGNC:566]	-0.04	4.91	4.71E-01
ENSG00000168610	STAT3	signal transducer and activator of transcription 3 [Source:HGNC Symbol;Acc:HGNC:11364]	-0.03	6.67	4.79E-01
ENSG00000165105	RASEF	RAS and EF-hand domain containing [Source:HGNC	-0.21	0.50	4.80E-01

		Symbol;Acc: HGNC:26464]			
ENSG00000117560	FASLG	Fas ligand [Source:HGNC Symbol;Acc: HGNC:11936]	-0.26	1.02	4.81E-01
ENSG00000115310	RTN4	reticulon 4 [Source:HGNC Symbol;Acc: HGNC:14085]	0.04	6.20	4.90E-01
ENSG00000033178	UBA6	ubiquitin like modifier activating enzyme 6 [Source:HGNC Symbol;Acc: HGNC:25581]	-0.03	5.57	4.93E-01
ENSG00000113328	CCNG1	cyclin G1 [Source:HGNC Symbol;Acc: HGNC:1592]	-0.03	7.42	4.98E-01
ENSG00000101384	JAG1	jagged 1 [Source:HGNC	-0.33	-0.72	4.99E-01

		Symbol;Acc:HGNC:6188]			
ENSG00000071967	CYBRD1	cytochrome b reductase 1 [Source:HGNC Symbol;Acc:HGNC:20797]	-0.25	-0.14	5.01E-01
ENSG00000083168	KAT6A	lysine acetyltransferase 6A [Source:HGNC Symbol;Acc:HGNC:13013]	-0.02	7.15	5.07E-01
ENSG00000090863	GLG1	golgi glycoprotein 1 [Source:HGNC Symbol;Acc:HGNC:4316]	0.02	7.47	5.11E-01
ENSG00000111328	CDK2AP1	cyclin dependent kinase 2 associated protein 1 [Source:HGNC Symbol;Acc:	0.15	1.66	5.14E-01

		HGNC:14002]			
ENSG00000198142	SOWAHC	sosondowah ankyrin repeat domain family member C [Source:HGNC Symbol;Acc:HGNC:26149]	0.18	1.61	5.16E-01
ENSG00000109654	TRIM2	tripartite motif containing 2 [Source:HGNC Symbol;Acc:HGNC:15974]	0.11	2.37	5.19E-01
ENSG00000112242	E2F3	E2F transcription factor 3 [Source:HGNC Symbol;Acc:HGNC:3115]	0.05	5.22	5.21E-01
ENSG00000159388	BTG2	BTG anti-proliferation factor 2 [Source:HGNC	-0.07	10.18	5.21E-01

		Symbol;Acc:HGNC:1131]			
ENSG00000138443	ABI2	abl interactor 2 [Source:HGNC Symbol;Acc:HGNC:24011]	-0.04	5.52	5.24E-01
ENSG00000169905	TOR1AIP2	torsin 1A interacting protein 2 [Source:HGNC Symbol;Acc:HGNC:24055]	0.03	3.96	5.28E-01
ENSG00000171940	**ZNF217**	zinc finger protein 217 [Source:HGNC Symbol;Acc:HGNC:13009]	0.03	6.68	5.29E-01
ENSG00000099940	SNAP29	synaptosome associated protein 29 [Source:HGNC Symbol;Acc:HGNC:11133]	-0.04	5.41	5.35E-01
ENSG00000116918	TSNAX	translin	0.02	5.44	5.36E-

		associated factor X [Source:HGNC Symbol;Acc:HGNC:12380]			01
ENSG00000198961	PJA2	praja ring finger ubiquitin ligase 2 [Source:HGNC Symbol;Acc:HGNC:17481]	0.03	6.98	5.41E-01
ENSG00000157933	**SKI**	SKI proto-oncogene [Source:HGNC Symbol;Acc:HGNC:10896]	0.05	7.14	5.44E-01
ENSG00000104866	PPP1R37	protein phosphatase 1 regulatory subunit 37 [Source:HGNC Symbol;Acc:HGNC:27607]	-0.04	3.62	5.53E-01

ENSG00000077684	JADE1	jade family PHD finger 1 [Source:HGNC Symbol;Acc:HGNC:30027]	-0.03	5.90	5.60E-01
ENSG00000143878	**RHOB**	ras homolog family member B [Source:HGNC Symbol;Acc:HGNC:668]	-0.19	5.49	5.62E-01
ENSG00000111266	DUSP16	dual specificity phosphatase 16 [Source:HGNC Symbol;Acc:HGNC:17909]	0.06	6.63	5.64E-01
ENSG00000135521	LTV1	LTV1 ribosome biogenesis factor [Source:HGNC Symbol;Acc:HGNC:21173]	0.03	6.53	5.65E-01
ENSG00000184743	ATL3	atlastin	0.03	5.11	5.65E-

		GTPase 3 [Source:HGNC Symbol;Acc:HGNC:24526]			01
ENSG00000163939	**PBRM1**	polybromo 1 [Source:HGNC Symbol;Acc:HGNC:30064]	-0.02	5.89	5.65E-01
ENSG00000151413	NUBPL	nucleotide binding protein like [Source:HGNC Symbol;Acc:HGNC:20278]	0.04	2.87	5.70E-01
ENSG00000163125	RPRD2	regulation of nuclear pre-mRNA domain containing 2 [Source:HGNC Symbol;Acc:HGNC:29039]	-0.02	6.12	5.70E-01
ENSG00000140396	NCOA2	nuclear receptor	0.03	5.86	5.75E-01

		coactivator 2 [Source:HGNC Symbol;Acc:HGNC:7669]			
ENSG00000165572	**KBTBD6**	kelch repeat and BTB domain containing 6 [Source:HGNC Symbol;Acc:HGNC:25340]	-0.05	2.93	5.77E-01
ENSG00000184007	PTP4A2	protein tyrosine phosphatase type IVA, member 2 [Source:HGNC Symbol;Acc:HGNC:9635]	0.03	8.12	5.77E-01
ENSG00000172175	MALT1	MALT1 paracaspase [Source:HGNC Symbol;Acc:HGNC:6819]	-0.02	7.30	5.77E-01
ENSG00000115159	GPD2	glycerol-3-phosphate dehydrogen	0.04	4.12	5.83E-01

		ase 2 [Source:HGNC Symbol;Acc:HGNC:4456]			
ENSG00000112697	TMEM30A	transmembrane protein 30A [Source:HGNC Symbol;Acc:HGNC:16667]	0.02	7.15	5.85E-01
ENSG00000083312	TNPO1	transportin 1 [Source:HGNC Symbol;Acc:HGNC:6401]	-0.02	6.04	5.86E-01
ENSG00000166483	WEE1	WEE1 G2 checkpoint kinase [Source:HGNC Symbol;Acc:HGNC:12761]	-0.09	2.26	5.90E-01
ENSG00000070759	TESK2	testis-specific kinase 2 [Source:HGNC Symbol;Acc:	0.05	3.95	5.91E-01

		HGNC:11732]			
ENSG00000153914	SREK1	splicing regulatory glutamic acid and lysine rich protein 1 [Source:HGNC Symbol;Acc:HGNC:17882]	0.02	6.57	5.92E-01
ENSG00000120137	PANK3	pantothenate kinase 3 [Source:HGNC Symbol;Acc:HGNC:19365]	0.02	6.15	5.95E-01
ENSG00000102401	ARMCX3	armadillo repeat containing, X-linked 3 [Source:HGNC Symbol;Acc:HGNC:24065]	-0.03	5.47	5.96E-01
ENSG00000173674	EIF1AX	eukaryotic translation initiation factor 1A, X-	-0.08	6.96	5.99E-01

		linked [Source:HGNC Symbol;Acc:HGNC:3250]			
ENSG00000257923	CUX1	cut like homeobox 1 [Source:HGNC Symbol;Acc:HGNC:2557]	0.03	4.27	5.99E-01
ENSG00000155744	**FAM126B**	family with sequence similarity 126 member B [Source:HGNC Symbol;Acc:HGNC:28593]	0.03	4.63	6.01E-01
ENSG00000123836	PFKFB2	6-phosphofructo-2-kinase/fructose-2,6-biphosphatase 2 [Source:HGNC Symbol;Acc:HGNC:8873]	-0.07	1.95	6.02E-01

ENSG00000112941	PAPD7	poly(A) RNA polymerase D7, non-canonical [Source:HGNC Symbol;Acc:HGNC:16705]	0.03	6.88	6.06E-01
ENSG00000148154	UGCG	UDP-glucose ceramide glucosyltransferase [Source:HGNC Symbol;Acc:HGNC:12524]	0.05	6.21	6.09E-01
ENSG00000155189	AGPAT5	1-acylglycerol-3-phosphate O-acyltransferase 5 [Source:HGNC Symbol;Acc:HGNC:20886]	0.03	4.39	6.14E-01
ENSG00000215301	DDX3X	DEAD-box helicase 3,	-0.07	9.08	6.16E-01

		X-linked [Source:HGNC Symbol;Acc:HGNC:2745]			
ENSG00000196937	**FAM3C**	family with sequence similarity 3 member C [Source:HGNC Symbol;Acc:HGNC:18664]	0.04	4.85	6.18E-01
ENSG00000162909	CAPN2	calpain 2 [Source:HGNC Symbol;Acc:HGNC:1479]	-0.04	7.66	6.19E-01
ENSG00000170348	TMED10	transmembrane p24 trafficking protein 10 [Source:HGNC Symbol;Acc:HGNC:16998]	-0.03	7.44	6.22E-01
ENSG00000177311	ZBTB38	zinc finger and BTB domain containing 38	-0.05	5.16	6.25E-01

		[Source:HGNC Symbol;Acc:HGNC:26636]			
ENSG00000039319	ZFYVE16	zinc finger FYVE-type containing 16 [Source:HGNC Symbol;Acc:HGNC:20756]	-0.02	4.16	6.28E-01
ENSG00000188215	DCUN1D3	defective in cullin neddylation 1 domain containing 3 [Source:HGNC Symbol;Acc:HGNC:28734]	-0.04	3.16	6.29E-01
ENSG00000144674	GOLGA4	golgin A4 [Source:HGNC Symbol;Acc:HGNC:4427]	0.03	5.91	6.32E-01
ENSG00000177051	FBXO46	F-box protein 46 [Source:HGNC	-0.05	5.26	6.35E-01

		Symbol;Acc: HGNC:25069]			
ENSG00000105856	HBP1	HMG-box transcription factor 1 [Source:HGNC Symbol;Acc: HGNC:23200]	0.03	6.72	6.41E-01
ENSG00000117868	ESYT2	extended synaptotagmin 2 [Source:HGNC Symbol;Acc: HGNC:22211]	0.03	8.09	6.42E-01
ENSG00000145332	KLHL8	kelch like family member 8 [Source:HGNC Symbol;Acc: HGNC:18644]	-0.03	4.04	6.50E-01
ENSG00000107771	CCSER2	coiled-coil serine rich protein 2 [Source:HGNC Symbol;Acc:	0.04	6.74	6.53E-01

		HGNC:29197]			
ENSG00000112425	EPM2A	epilepsy, progressive myoclonus type 2A, Lafora disease (laforin) [Source:HGNC Symbol;Acc:HGNC:3413]	0.05	3.67	6.56E-01
ENSG00000120805	ARL1	ADP ribosylation factor like GTPase 1 [Source:HGNC Symbol;Acc:HGNC:692]	0.02	5.09	6.59E-01
ENSG00000184602	SNN	stannin [Source:HGNC Symbol;Acc:HGNC:11149]	0.06	6.03	6.63E-01
ENSG00000130449	ZSWIM6	zinc finger SWIM-type containing 6 [Source:HGNC Symbol;Acc:	0.03	5.09	6.64E-01

		HGNC:29316]			
ENSG00000175582	RAB6A	RAB6A, member RAS oncogene family [Source:HGNC Symbol;Acc:HGNC:9786]	-0.02	6.18	6.65E-01
ENSG00000076706	MCAM	melanoma cell adhesion molecule [Source:HGNC Symbol;Acc:HGNC:6934]	-0.13	0.02	6.66E-01
ENSG00000118579	MED28	mediator complex subunit 28 [Source:HGNC Symbol;Acc:HGNC:24628]	-0.02	6.64	6.66E-01
ENSG00000091009	RBM27	RNA binding motif protein 27 [Source:HGNC Symbol;Acc:	-0.02	5.91	6.67E-01

		HGNC:29243]			
ENSG00000153561	RMND5A	required for meiotic nuclear division 5 homolog A [Source:HGNC Symbol;Acc:HGNC:25850]	0.02	5.69	6.75E-01
ENSG00000155096	AZIN1	antizyme inhibitor 1 [Source:HGNC Symbol;Acc:HGNC:16432]	0.02	6.37	6.76E-01
ENSG00000120889	TNFRSF10B	TNF receptor superfamily member 10b [Source:HGNC Symbol;Acc:HGNC:11905]	-0.03	6.27	6.77E-01
ENSG00000101972	**STAG2**	stromal antigen 2 [Source:HGNC	0.01	6.93	6.78E-01

		Symbol;Acc:HGNC:11355]			
ENSG00000100485	SOS2	SOS Ras/Rho guanine nucleotide exchange factor 2 [Source:HGNC Symbol;Acc:HGNC:11188]	0.02	5.66	6.79E-01
ENSG00000135655	USP15	ubiquitin specific peptidase 15 [Source:HGNC Symbol;Acc:HGNC:12613]	0.02	7.12	6.87E-01
ENSG00000185129	PURA	purine rich element binding protein A [Source:HGNC Symbol;Acc:HGNC:9701]	-0.03	5.03	6.99E-01
ENSG00000148143	ZNF462	zinc finger protein 462	0.16	-0.06	7.08E-01

		[Source:HGNC Symbol;Acc:HGNC:21684]			
ENSG00000183735	TBK1	TANK binding kinase 1 [Source:HGNC Symbol;Acc:HGNC:11584]	-0.05	4.73	7.11E-01
ENSG00000122707	RECK	reversion inducing cysteine rich protein with kazal motifs [Source:HGNC Symbol;Acc:HGNC:11345]	-0.02	4.99	7.16E-01
ENSG00000122566	HNRNPA2B1	heterogeneous nuclear ribonucleoprotein A2/B1 [Source:HGNC Symbol;Acc:HGNC:5033]	0.01	9.69	7.17E-01

ENSG00000113368	LMNB1	lamin B1 [Source:HGNC Symbol;Acc:HGNC:6637]	0.03	5.03	7.17E-01
ENSG00000107560	RAB11FIP2	RAB11 family interacting protein 2 [Source:HGNC Symbol;Acc:HGNC:29152]	0.02	5.72	7.17E-01
ENSG00000130638	ATXN10	ataxin 10 [Source:HGNC Symbol;Acc:HGNC:10549]	0.02	5.80	7.22E-01
ENSG00000095015	**MAP3K1**	mitogen-activated protein kinase kinase kinase 1 [Source:HGNC Symbol;Acc:HGNC:6848]	0.03	8.20	7.24E-01
ENSG00000171150	SOCS5	suppressor of cytokine signaling 5	-0.02	4.54	7.31E-01

		[Source:HGNC Symbol;Acc:HGNC:16852]			
ENSG00000125633	CCDC93	coiled-coil domain containing 93 [Source:HGNC Symbol;Acc:HGNC:25611]	0.01	6.25	7.34E-01
ENSG00000029993	HMGB3	high mobility group box 3 [Source:HGNC Symbol;Acc:HGNC:5004]	0.11	0.70	7.37E-01
ENSG00000115808	STRN	striatin [Source:HGNC Symbol;Acc:HGNC:11424]	0.01	5.74	7.54E-01
ENSG00000149792	MRPL49	mitochondrial ribosomal protein L49 [Source:HGNC Symbol;Acc:	0.02	5.86	7.57E-01

		HGNC:1176]			
ENSG00000160213	CSTB	cystatin B [Source:HGNC Symbol;Acc:HGNC:2482]	0.03	5.96	7.57E-01
ENSG00000100811	YY1	YY1 transcription factor [Source:HGNC Symbol;Acc:HGNC:12856]	0.01	7.09	7.59E-01
ENSG00000100345	MYH9	myosin heavy chain 9 [Source:HGNC Symbol;Acc:HGNC:7579]	-0.02	9.63	7.59E-01
ENSG00000099194	SCD	stearoyl-CoA desaturase [Source:HGNC Symbol;Acc:HGNC:10571]	0.11	1.11	7.69E-01
ENSG00000102144	PGK1	phosphoglycerate kinase 1 [Source:HG	0.01	7.52	7.74E-01

		NC Symbol;Acc: HGNC:8896]			
ENSG00000163602	RYBP	RING1 and YY1 binding protein [Source:HGNC Symbol;Acc: HGNC:10480]	-0.02	5.95	7.81E-01
ENSG00000113300	**CNOT6**	CCR4-NOT transcription complex subunit 6 [Source:HGNC Symbol;Acc: HGNC:14099]	0.02	5.64	7.85E-01
ENSG00000107099	DOCK8	dedicator of cytokinesis 8 [Source:HGNC Symbol;Acc: HGNC:19191]	-0.03	7.03	7.87E-01
ENSG00000136100	VPS36	vacuolar protein sorting 36 homolog [Source:HG	0.01	6.53	7.87E-01

		NC Symbol;Acc: HGNC:20312]			
ENSG00000026508	CD44	CD44 molecule (Indian blood group) [Source:HGNC Symbol;Acc: HGNC:1681]	0.01	8.30	7.91E-01
ENSG00000170677	SOCS6	suppressor of cytokine signaling 6 [Source:HGNC Symbol;Acc: HGNC:16833]	0.07	1.04	7.91E-01
ENSG00000120709	FAM53C	family with sequence similarity 53 member C [Source:HGNC Symbol;Acc: HGNC:1336]	-0.02	4.73	7.97E-01
ENSG00000138663	COPS4	COP9 signalosome subunit 4 [Source:HG	-0.01	4.95	7.98E-01

		NC Symbol;Acc: HGNC:16702]			
ENSG00000101266	CSNK2A1	casein kinase 2 alpha 1 [Source:HGNC Symbol;Acc: HGNC:2457]	-0.01	6.15	7.99E-01
ENSG00000145495	MARCH6	membrane associated ring-CH-type finger 6 [Source:HGNC Symbol;Acc: HGNC:30550]	0.01	7.97	8.01E-01
ENSG00000156642	NPTN	neuroplastin [Source:HGNC Symbol;Acc: HGNC:17867]	0.02	5.93	8.03E-01
ENSG00000198642	KLHL9	kelch like family member 9 [Source:HGNC	0.02	5.32	8.03E-01

		Symbol;Acc: HGNC:18732]			
ENSG00000140443	IGF1R	insulin like growth factor 1 receptor [Source:HGNC Symbol;Acc: HGNC:5465]	0.04	5.68	8.13E-01
ENSG00000075420	FNDC3B	fibronectin type III domain containing 3B [Source:HGNC Symbol;Acc: HGNC:24670]	0.02	3.71	8.15E-01
ENSG00000067955	CBFB	core-binding factor beta subunit [Source:HGNC Symbol;Acc: HGNC:1539]	-0.01	6.92	8.16E-01
ENSG00000136868	SLC31A1	solute carrier family 31 member 1	0.03	2.77	8.16E-01

		[Source:HGNC Symbol;Acc:HGNC:11016]			
ENSG00000196268	ZNF493	zinc finger protein 493 [Source:HGNC Symbol;Acc:HGNC:23708]	0.02	4.63	8.19E-01
ENSG00000197312	DDI2	DNA damage inducible 1 homolog 2 [Source:HGNC Symbol;Acc:HGNC:24578]	0.01	5.28	8.21E-01
ENSG00000001631	KRIT1	KRIT1, ankyrin repeat containing [Source:HGNC Symbol;Acc:HGNC:1573]	-0.01	5.68	8.22E-01
ENSG00000170802	FOXN2	forkhead box N2 [Source:HGNC	0.02	6.48	8.26E-01

		Symbol;Acc: HGNC:5281]			
ENSG00000116199	FAM20B	family with sequence similarity 20 member B [Source:HGNC Symbol;Acc: HGNC:23017]	0.01	5.32	8.27E-01
ENSG00000108510	MED13	mediator complex subunit 13 [Source:HGNC Symbol;Acc: HGNC:22474]	0.01	6.02	8.31E-01
ENSG00000163848	ZNF148	zinc finger protein 148 [Source:HGNC Symbol;Acc: HGNC:12933]	0.01	5.56	8.32E-01
ENSG00000106723	SPIN1	spindlin 1 [Source:HGNC Symbol;Acc: HGNC:11243]	-0.01	5.84	8.34E-01
ENSG00000144445	KANSL1L	KAT8	-	3.22	8.38E-

		regulatory NSL complex subunit 1 like [Source:HGNC Symbol;Acc:HGNC:26310]	0.03		01
ENSG00000128923	**FAM63B**	family with sequence similarity 63 member B [Source:HGNC Symbol;Acc:HGNC:26954]	0.02	4.99	8.39E-01
ENSG00000175130	MARCKSL1	MARCKS like 1 [Source:HGNC Symbol;Acc:HGNC:7142]	-0.02	6.60	8.45E-01
ENSG00000094916	CBX5	chromobox 5 [Source:HGNC Symbol;Acc:HGNC:1555]	0.01	6.29	8.47E-01
ENSG00000183044	ABAT	4-aminobutyr	0.01	3.40	8.48E-01

		ate aminotransferase [Source:HGNC Symbol;Acc:HGNC:23]			
ENSG00000013375	PGM3	phosphoglucomutase 3 [Source:HGNC Symbol;Acc:HGNC:8907]	0.01	4.44	8.49E-01
ENSG00000074201	CLNS1A	chloride nucleotide-sensitive channel 1A [Source:HGNC Symbol;Acc:HGNC:2080]	0.01	6.16	8.49E-01
ENSG00000169398	PTK2	protein tyrosine kinase 2 [Source:HGNC Symbol;Acc:HGNC:9611]	0.03	2.26	8.49E-01
ENSG00000108256	NUFIP2	NUFIP2, FMR1 interacting protein 2 [Source:HG	-0.01	7.11	8.64E-01

		NC Symbol;Acc: HGNC:17634]			
ENSG00000163283	ALPP	alkaline phosphatase, placental [Source:HGNC Symbol;Acc: HGNC:439]	-0.05	0.81	8.68E-01
ENSG00000132294	EFR3A	EFR3 homolog A [Source:HGNC Symbol;Acc: HGNC:28970]	0.01	6.14	8.70E-01
ENSG00000138069	RAB1A	RAB1A, member RAS oncogene family [Source:HGNC Symbol;Acc: HGNC:9758]	-0.01	6.55	8.73E-01
ENSG00000065183	WDR3	WD repeat domain 3 [Source:HGNC Symbol;Acc: HGNC:1275	-0.01	5.29	8.73E-01

		5]			
ENSG00000148835	TAF5	TATA-box binding protein associated factor 5 [Source:HGNC Symbol;Acc:HGNC:11539]	-0.02	4.19	8.75E-01
ENSG00000119669	IRF2BPL	interferon regulatory factor 2 binding protein like [Source:HGNC Symbol;Acc:HGNC:14282]	-0.02	5.08	8.79E-01
ENSG00000135535	CD164	CD164 molecule [Source:HGNC Symbol;Acc:HGNC:1632]	-0.01	7.54	8.80E-01
ENSG00000105887	MTPN	myotrophin [Source:HGNC Symbol;Acc:HGNC:15667]	-0.01	7.32	8.82E-01

ENSG00000131791	PRKAB2	protein kinase AMP-activated non-catalytic subunit beta 2 [Source:HGNC Symbol;Acc:HGNC:9379]	0.01	5.80	8.84E-01
ENSG00000056586	RC3H2	ring finger and CCCH-type domains 2 [Source:HGNC Symbol;Acc:HGNC:21461]	0.01	6.01	8.86E-01
ENSG00000057757	PITHD1	PITH domain containing 1 [Source:HGNC Symbol;Acc:HGNC:25022]	-0.01	6.57	8.87E-01
ENSG00000113387	SUB1	SUB1 homolog, transcriptional regulator	-0.01	6.56	8.89E-01

		[Source:HGNC Symbol;Acc:HGNC:19985]			
ENSG00000048544	MRPS10	mitochondrial ribosomal protein S10 [Source:HGNC Symbol;Acc:HGNC:14502]	-0.01	5.78	8.91E-01
ENSG00000138081	**FBXO11**	F-box protein 11 [Source:HGNC Symbol;Acc:HGNC:13590]	0.00	6.64	8.93E-01
ENSG00000112902	SEMA5A	semaphorin 5A [Source:HGNC Symbol;Acc:HGNC:10736]	-0.04	1.13	8.98E-01
ENSG00000083099	LYRM2	LYR motif containing 2 [Source:HGNC Symbol;Acc:HGNC:2522	-0.01	3.78	9.01E-01

		9]			
ENSG00000122335	SERAC1	serine active site containing 1 [Source:HGNC Symbol;Acc:HGNC:21061]	-0.01	2.92	9.08E-01
ENSG00000155962	CLIC2	chloride intracellular channel 2 [Source:HGNC Symbol;Acc:HGNC:2063]	-0.03	0.90	9.22E-01
ENSG00000162607	USP1	ubiquitin specific peptidase 1 [Source:HGNC Symbol;Acc:HGNC:12607]	0.01	6.53	9.27E-01
ENSG00000170242	USP47	ubiquitin specific peptidase 47 [Source:HGNC Symbol;Acc:HGNC:20076]	0.00	6.01	9.27E-01

ENSG00000102554	KLF5	Kruppel like factor 5 [Source:HGNC Symbol;Acc:HGNC:6349]	-0.02	2.89	9.34E-01
ENSG00000142634	EFHD2	EF-hand domain family member D2 [Source:HGNC Symbol;Acc:HGNC:28670]	-0.02	6.91	9.37E-01
ENSG00000148730	**EIF4EBP2**	eukaryotic translation initiation factor 4E binding protein 2 [Source:HGNC Symbol;Acc:HGNC:3289]	0.00	7.12	9.40E-01
ENSG00000166681	BEX3	brain expressed X-linked 3 [Source:HGNC Symbol;Acc:HGNC:13388]	0.01	5.63	9.49E-01

ENSG00000111647	UHRF1BP1L	UHRF1 binding protein 1 like [Source:HGNC Symbol;Acc:HGNC:29102]	0.00	4.33	9.52E-01
ENSG00000085788	DDHD2	DDHD domain containing 2 [Source:HGNC Symbol;Acc:HGNC:29106]	0.00	5.68	9.52E-01
ENSG00000147548	WHSC1L1	Wolf-Hirschhorn syndrome candidate 1-like 1 [Source:HGNC Symbol;Acc:HGNC:12767]	0.00	6.89	9.57E-01
ENSG00000070882	OSBPL3	oxysterol binding protein like 3 [Source:HGNC	0.00	5.73	9.61E-01

		Symbol;Acc:HGNC:16370]			
ENSG00000059728	MXD1	MAX dimerization protein 1 [Source:HGNC Symbol;Acc:HGNC:6761]	0.00	4.36	9.68E-01
ENSG00000100647	SUSD6	sushi domain containing 6 [Source:HGNC Symbol;Acc:HGNC:19956]	0.00	6.55	9.70E-01
ENSG00000123684	LPGAT1	lysophosphatidylglycerol acyltransferase 1 [Source:HGNC Symbol;Acc:HGNC:28985]	0.00	5.45	9.76E-01
ENSG00000161813	LARP4	La ribonucleoprotein domain family	0.00	4.77	9.79E-01

		member 4 [Source:HGNC Symbol;Acc:HGNC:24320]			
ENSG00000114098	ARMC8	armadillo repeat containing 8 [Source:HGNC Symbol;Acc:HGNC:24999]	0.00	5.17	9.83E-01
ENSG00000177125	ZBTB34	zinc finger and BTB domain containing 34 [Source:HGNC Symbol;Acc:HGNC:31446]	0.00	3.83	9.84E-01
ENSG00000011405	PIK3C2A	phosphatidylinositol-4-phosphate 3-kinase catalytic subunit type 2 alpha [Source:HGNC	0.00	5.43	9.95E-01

		Symbol;Acc: HGNC:8971]			
ENSG00000123091	RNF11	ring finger protein 11 [Source:HGNC Symbol;Acc: HGNC:10056]	0.00	6.45	9.96E-01
ENSG00000018189	RUFY3	RUN and FYVE domain containing 3 [Source:HGNC Symbol;Acc: HGNC:30285]	0.00	4.73	9.96E-01
ENSG00000134198	TSPAN2	tetraspanin 2 [Source:HGNC Symbol;Acc: HGNC:20659]	0.00	4.10	9.99E-01
ENSG00000172575	**RASGRP1**	RAS guanyl releasing protein 1 [Source:HGNC Symbol;Acc: HGNC:9878]	0.00	8.41	9.99E-01
ENSG00000159399	HK2	hexokinase	0.00	3.94	1.00E+0

		2 [Source:HGNC Symbol;Acc:HGNC:4923]		0
ENSG00000013588	GPRC5A	G protein-coupled receptor class C group 5 member A [Source:HGNC Symbol;Acc:HGNC:9836]	-	
ENSG00000064042	LIMCH1	LIM and calponin homology domains 1 [Source:HGNC Symbol;Acc:HGNC:29191]	-	
ENSG00000078399	HOXA9	homeobox A9 [Source:HGNC Symbol;Acc:HGNC:5109]	-	
ENSG00000079308	TNS1	tensin 1 [Source:HGNC	-	

		Symbol;Acc: HGNC:11973]	
ENSG00000092969	TGFB2	transforming growth factor beta 2 [Source:HGNC Symbol;Acc: HGNC:11768]	-
ENSG00000102221	JADE3	jade family PHD finger 3 [Source:HGNC Symbol;Acc: HGNC:22982]	-
ENSG00000104369	JPH1	junctophilin 1 [Source:HGNC Symbol;Acc: HGNC:14201]	-
ENSG00000111799	COL12A1	collagen type XII alpha 1 chain [Source:HGNC Symbol;Acc:	-

		HGNC:2188]	
ENSG00000113594	LIFR	leukemia inhibitory factor receptor alpha [Source:HGNC Symbol;Acc:HGNC:6597]	-
ENSG00000114757	PEX5L	peroxisomal biogenesis factor 5 like [Source:HGNC Symbol;Acc:HGNC:30024]	-
ENSG00000115844	DLX2	distal-less homeobox 2 [Source:HGNC Symbol;Acc:HGNC:2915]	-
ENSG00000116132	PRRX1	paired related homeobox 1 [Source:HGNC Symbol;Acc:HGNC:9142]	-

ENSG00000118946	PCDH17	protocadherin 17 [Source:HGNC Symbol;Acc:HGNC:14267]	-
ENSG00000122420	PTGFR	prostaglandin F receptor [Source:HGNC Symbol;Acc:HGNC:9600]	-
ENSG00000122778	KIAA1549	KIAA1549 [Source:HGNC Symbol;Acc:HGNC:22219]	-
ENSG00000122786	CALD1	caldesmon 1 [Source:HGNC Symbol;Acc:HGNC:1441]	-
ENSG00000125398	SOX9	SRY-box 9 [Source:HGNC Symbol;Acc:HGNC:11204]	-
ENSG00000135116	HRK	harakiri, BCL2	-

		interacting protein [Source:HGNC Symbol;Acc:HGNC:5185]	
ENSG00000135378	PRRG4	proline rich and Gla domain 4 [Source:HGNC Symbol;Acc:HGNC:30799]	-
ENSG00000135862	LAMC1	laminin subunit gamma 1 [Source:HGNC Symbol;Acc:HGNC:6492]	-
ENSG00000136634	IL10	interleukin 10 [Source:HGNC Symbol;Acc:HGNC:5962]	-
ENSG00000138639	ARHGAP24	Rho GTPase activating protein 24 [Source:HGNC Symbol;Acc:	-

		HGNC:25361]	
ENSG00000143867	OSR1	odd-skipped related transciption factor 1 [Source:HGNC Symbol;Acc:HGNC:8111]	-
ENSG00000151090	THRB	thyroid hormone receptor beta [Source:HGNC Symbol;Acc:HGNC:11799]	-
ENSG00000152078	TMEM56	transmembrane protein 56 [Source:HGNC Symbol;Acc:HGNC:26477]	-
ENSG00000153904	DDAH1	dimethylarginine dimethylaminohydrolase 1 [Source:HG	-

		NC Symbol;Acc: HGNC:2715]	
ENSG00000154380	ENAH	enabled homolog (Drosophila) [Source:HGNC Symbol;Acc: HGNC:18271]	-
ENSG00000159217	IGF2BP1	insulin like growth factor 2 mRNA binding protein 1 [Source:HGNC Symbol;Acc: HGNC:28866]	-
ENSG00000162407	PLPP3	phospholipid phosphatase 3 [Source:HGNC Symbol;Acc: HGNC:9229]	-
ENSG00000162409	PRKAA2	protein kinase AMP-activated	-

		catalytic subunit alpha 2 [Source:HGNC Symbol;Acc:HGNC:9377]	
ENSG00000163293	NIPAL1	NIPA like domain containing 1 [Source:HGNC Symbol;Acc:HGNC:27194]	-
ENSG00000163637	PRICKLE2	prickle planar cell polarity protein 2 [Source:HGNC Symbol;Acc:HGNC:20340]	-
ENSG00000164176	EDIL3	EGF like repeats and discoidin domains 3 [Source:HGNC Symbol;Acc:HGNC:3173]	-
ENSG00000164761	TNFRSF11B	TNF	-

		receptor superfamily member 11b [Source:HGNC Symbol;Acc:HGNC:11909]	
ENSG00000165092	ALDH1A1	aldehyde dehydrogenase 1 family member A1 [Source:HGNC Symbol;Acc:HGNC:402]	-
ENSG00000166444	ST5	suppression of tumorigenicity 5 [Source:HGNC Symbol;Acc:HGNC:11350]	-
ENSG00000169245	CXCL10	C-X-C motif chemokine ligand 10 [Source:HGNC Symbol;Acc:HGNC:1063	-

		7]	
ENSG00000171714	ANO5	anoctamin 5 [Source:HGNC Symbol;Acc:HGNC:27337]	-
ENSG00000172380	GNG12	G protein subunit gamma 12 [Source:HGNC Symbol;Acc:HGNC:19663]	-
ENSG00000173391	OLR1	oxidized low density lipoprotein receptor 1 [Source:HGNC Symbol;Acc:HGNC:8133]	-
ENSG00000176597	B3GNT5	UDP-GlcNAc:betaGal beta-1,3-N-acetylglucosaminyltransferase 5 [Source:HGNC Symbol;Acc:	-

		HGNC:15684]	
ENSG00000180592	SKIDA1	SKI/DACH domain containing 1 [Source:HGNC Symbol;Acc:HGNC:32697]	-
ENSG00000181449	SOX2	SRY-box 2 [Source:HGNC Symbol;Acc:HGNC:11195]	-
ENSG00000182752	PAPPA	pappalysin 1 [Source:HGNC Symbol;Acc:HGNC:8602]	-
ENSG00000186205	MARC1	mitochondrial amidoxime reducing component 1 [Source:HGNC Symbol;Acc:HGNC:26189]	-

ENSG00000203727	SAMD5	sterile alpha motif domain containing 5 [Source:HGNC Symbol;Acc:HGNC:21180]	-
ENSG00000206075	SERPINB5	serpin family B member 5 [Source:HGNC Symbol;Acc:HGNC:8949]	-
ENSG00000214736	TOMM6	translocase of outer mitochondrial membrane 6 [Source:HGNC Symbol;Acc:HGNC:34528]	-
ENSG00000222014	RAB6C	RAB6C, member RAS oncogene family [Source:HG	-

		NC Symbol;Acc: HGNC:16525]	
ENSG00000263001	GTF2I	general transcription factor IIi [Source:HGNC Symbol;Acc: HGNC:4659]	-
ENSG00000272602	ZNF595	zinc finger protein 595 [Source:HGNC Symbol;Acc: HGNC:27196]	-
